# Multiomic assessments of LNCaP and derived cell strains reveal determinants of prostate cancer pathobiology

**DOI:** 10.1172/JCI194727

**Published:** 2025-09-16

**Authors:** Arnab Bose, Armand Bankhead, Ilsa Coleman, Thomas Persse, Wanting Han, Patricia Galipeau, Brian Hanratty, Tony Chu, Jared Lucas, Dapei Li, Rabeya Bilkis, Pushpa Itagi, Sajida Hassan, Mallory Beightol, Minjeong Ko, Ruth Dumpit, Michael Haffner, Colin Pritchard, Gavin Ha, Peter S. Nelson

**Affiliations:** 1 Divisions of Human Biology and; 2 Public Health Sciences, Fred Hutchinson Cancer Center, Seattle, Washington, USA.; 3 Department of Laboratory Medicine and Pathology, University of Washington, Seattle, Washington, USA.; 4 Division of Clinical Research, Fred Hutchinson Cancer Center, Seattle, Washington, USA.

**Keywords:** Cell biology, Oncology, Epigenetics, Molecular pathology, Prostate cancer

## Abstract

A cornerstone of research to improve cancer outcomes involves studies of model systems to identify causal drivers of oncogenesis, understand mechanisms leading to metastases, and develop new therapeutics. Although most cancer types are represented by large cell line panels that reflect diverse neoplastic genotypes and phenotypes found in patients, prostate cancer is notable for a very limited repertoire of models that recapitulate the pathobiology of human disease. Of these, the lymph node carcinoma of the prostate (LNCaP) cell line has served as the major resource for basic and translational studies. Here, we delineated the molecular composition of LNCaP and multiple substrains through analyses of whole-genome sequences, transcriptomes, chromatin structure, androgen receptor (AR) cistromes, and functional studies. Our results determined that LNCaP exhibits substantial subclonal diversity, ongoing genomic instability, and phenotype plasticity. Several oncogenic features were consistently present across strains, but others were unexpectedly variable, such as ETV1 expression, Y chromosome loss, a reliance on WNT and glucocorticoid receptor activity, and distinct AR alterations maintaining AR pathway activation. These results document the inherent molecular heterogeneity and ongoing genomic instability that drive diverse prostate cancer phenotypes and provide a foundation for the accurate interpretation and reproduction of research findings.

## Abstract 2

The study applied genome-scale multiomics approaches to detail the subclonal diversity, genomic instability and phenotypic plasticity of the human LNCaP model used widely for understanding the pathobiology of prostate cancer.

## Introduction

Cancer models serve as essential resources for understanding oncogenic processes and developing therapeutics. To this end, models that accurately recapitulate key biological features of human pathogenesis are required. Carcinoma of the prostate is an extraordinarily common malignancy that accounts for substantial morbidity and mortality worldwide (1). The vast majority of localized and metastatic prostate carcinomas depend on androgenic hormones and signaling through the androgen receptor (AR) to maintain survival and growth, a feature that has served as a therapeutic focal point for more than 70 years (2, 3). However, prostate carcinoma research has been challenged by the difficulty of propagating prostate carcinomas ex vivo that retain AR signaling and the attendant neoplastic phenotypes that are commonly observed in patients (4). Consequently, the field has relied extensively on a very small number of cell lines and their derivatives as surrogates for different stages of prostate carcinoma progression, metastasis, and treatment resistance (5, 6).

The lymph node carcinoma of the prostate (LNCaP) cell line is the most extensively characterized and utilized model system in prostate carcinoma research, with more than 10,000 citations in the published literature as of 2025. First described in 1980 and more extensively characterized in 1983, LNCaP was established from a needle biopsy of a left supraclavicular lymph node from a 50-year-old White man (7, 8). Notable features of LNCaP cells include AR expression, androgen-regulated secretion of proteins produced by luminal prostate epithelium including prostate-specific antigen (PSA), proliferation influenced by exogenous androgenic hormones, robust growth in vitro using standard cell culture methods, and the ability to form tumors in immunocompromised mice (7–10). Of relevance, the *AR* gene in LNCaP is mutated at the ligand binding domain (LBD) (T878A) that allows certain anti-androgens, progesterone, or estradiol to act as functional agonists (11). From the original culture, several substrains were established, including a fast-growing colony (LNCaP_FGC) that is now the de facto standard model available from cell line repositories such as American Type Culture Collection (ATCC) (8).

An important but underappreciated attribute of LNCaP concerns the substantial genomic heterogeneity of both the original LNCaP isolate and the current LNCaP_FGC line that were never subjected to clonal selection. This feature, coupled with the inherent DNA mismatch-repair deficiency and resulting genomic instability of LNCaP, has produced a remarkable spectrum of substrains established through propagation in specific culture conditions, passaging in murine hosts, and exposures to therapeutics, particularly those inhibiting AR signaling (8, 10, 12–17). As treatments for advanced prostate carcinoma have evolved, the flexibility of the LNCaP model has been iteratively exploited to ascertain mechanisms driving resistance and used to develop new therapeutics that have extended patient survival (13, 18–21). LNCaP has served as a remarkably versatile prostate carcinoma model, but the molecular characteristics of several important substrains have not been established nor compared in a systematic manner. In this study, we used genome-scale approaches to extensively characterize the genomic, transcriptomic, cistromic, and epigenetic features of the standard LNCaP_FGC line, and a spectrum of 12 derivative models that have been employed for studies of prostate carcinoma. The results provide insights into the diverse biochemical characteristics that endow prostate carcinoma with the ability to survive AR-directed therapy and identify features that underlie variation in treatment-resistant phenotypes observed in patients. Importantly, the results also emphasize the importance of recognizing the inherent heterogeneity and genomic instability of LNCaP_FGC and derivative strains that require careful experimental design to ensure reproducibility and accurate interpretation of results when using these models for studies of prostate carcinoma pathobiology.

## Results

### The LNCaP_FGC genome.

The FGC subline of the original LNCaP cell culture is the current standard LNCaP cell line available from ATCC to investigators. To identify alterations in the genome of the LNCaP_FGC line, we performed whole-genome sequencing (WGS) on LNCaP_FGC cells obtained directly from ATCC, listed as CRL-1740 (lot 70042941), generating 1.39 billion reads with a 99.9% alignment rate and mean coverage of 40× (see Methods). Because germline DNA from the patient from whom the LNCaP line was derived is not available as a reference, we compared the WGS results against dbSNP130 to identify LNCaP_FGC features that were not reported as common germline variants.

**Figure 1:**
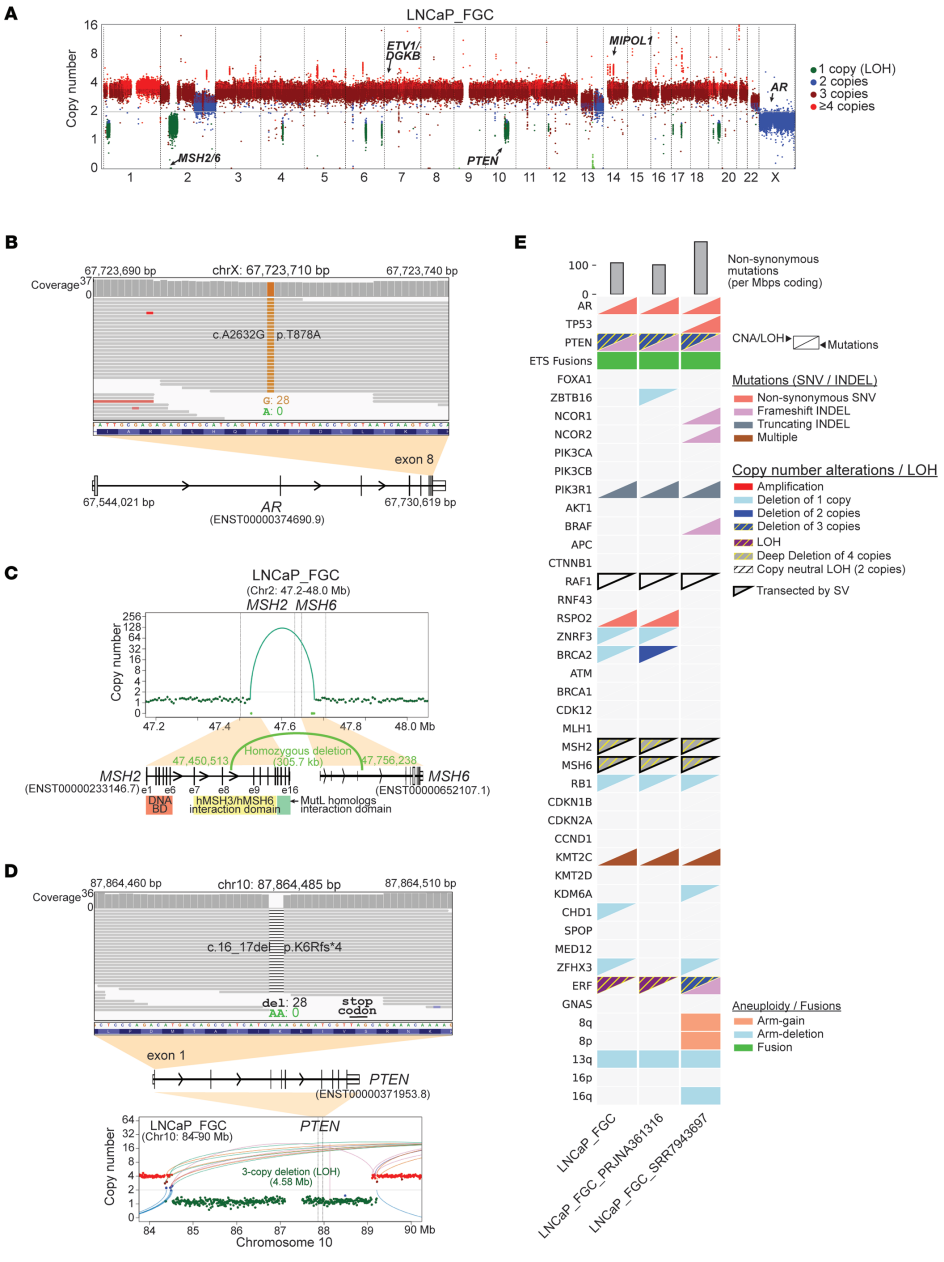
The LNCaP_FGC genome comprises major oncogenic events observed in metastatic prostate cancer. (**A**) Integer copy number profile for LNCaP parental line (FGC) whole-genome sequencing (WGS) data. Coloring represents estimated copy number: deletions (green), copy neutral (blue), gain (dark red), and amplification (bright red). Key genes are annotated on the plot based on corresponding copy number. (**B**) WGS reads visualized using Integrated Genomics Viewer (IGV) for an observed SNV in exon 8 of the AR gene in LNCaP_FGC. (**C**) Integer copy number profile showing a deletion structural rearrangement event in LNCaP_FGC for the genomic region encompassing genes *MSH2* and *MSH6*, with an observed homozygous deletion between the 2 genes. (**D**) WGS reads visualized using IGV for an observed frameshift deletion in *PTEN* in LNCaP_FGC. The copy number profile shows a deletion of the region in chromosome 10 that encompasses PTEN, resulting in 2 copy loss. (**E**) Mutation and copy number alteration (CNA) status for selected genes with recurrent alterations in metastatic prostate cancer. The bottom right triangle indicates an observed pathogenic mutation (SNV/INDEL) within the gene; top left triangle indicates CNA status (amplification, shallow deletions, deletions, deep deletions) that overlap the gene. CNA events that also have loss of heterozygosity (LOH) status are depicted with a hatch pattern. “Multiple” indicates instances where 2+ pathogenic mutations are observed for that gene. Tumor mutation burden was computed as the number of nonsynonymous mutations per megabase pairs of coding regions (top). Aneuploidy status (arm gain, arm deletion) is indicated for select chromosome arms. Fusion status indicates evidence for genomic rearrangement involving an E26 transformation specific (ETS) transcription factor. Genes that have been transected by at least 1 of 2 breakpoints of a structural variation (SV) event are indicated with a black border around the triangle. INDEL, insertions/deletions; SNV, single nucleotide variant.

Relevant metrics for the LNCaP_FGC genome are the following: 409,210 single nucleotide variants (SNVs; 134.46 mutations/Mb across the genome) with an estimated tumor ploidy of 3.2, consistent with a mixture of diploid (2N) and tetraploid (4N) cell populations (7, 22, 23). Within 25,840,698 nucleotides of coding sequence, there were 4,224 SNVs (163.46 mutations/Mb coding sequence) and 456 insertions/deletions, of which 362 exhibited features of pathogenic alterations such as stop-gain events (Supplemental Table 1; supplemental material available online with this article; https://doi.org/10.1172/JCI194727DS1). We characterized structural DNA alterations in the LNCaP_FGC cells and determined that 27% of the genome was altered by a copy loss or gain event (Figure 1A and Supplemental Tables 1 and 2). Large deletions (arm level) occurred in chromosomes 2q and 13. Using a cut-point of 10 kbps as a minimum size criterion, there were 321 structural rearrangements identified, including 58 chromosomal deletions, 36 duplications, 54 inversions, and 172 interchromosomal translocations (Supplemental Tables 1 and 2).

Analyses of the LNCaP_FGC genome confirmed prior studies identifying 4 major oncogenic events that are considered recurrent driver alterations in metastatic prostate carcinomas (24) (Figure 1, A–E, Supplemental Figure 1A, and Supplemental Tables 2 and 3): (a) a single base substitution involving T878A in the of the AR (11, 25); (b) a complex gene rearrangement of *MIPOL1*-*DGKB* that involves the E26 transformation-specific (ETS) family oncogene *ETV1* (26); (c) biallelic loss of the *PTEN* tumor suppressor (27, 28); and (d) a gene rearrangement event that results in the disruption of both *MSH2* and *MSH6* genes that mediate DNA mismatch-repair processes associated with a hypermutated genome (29, 30).

In addition, we determined that LNCaP_FGC harbors genomic alterations in several other genes with the potential to influence neoplastic phenotypes, as shown in Figure 1E, Supplemental Figure 1, B and C, and Supplemental Table 3: (a) monoallelic loss of *RB1*; (b) biallelic inactivation of *KMT2C* by frameshift deletion and a missense mutation (31); (c) deletion and LOH of the ETS family member *ERF* (*ETS2 Repressor Factor*) (32); (d) a monoallelic truncating mutation in *PIK3R1* (p.R639*), potentially leading to AKT activation (33); (e) a nonsynonymous mutation in CHEK2 (c.1160C>T/p.Thr387Asn), which has been shown to impair CHEK2 autophosphorylation and activation (34, 35); (f) a translocation involving the *RAF1* oncogene (36); (g) a mutation in *HOXB13* (c431A>C/p.L144P) located in the conserved functional domain predicted to be deleterious (37); (h) a pathogenic variant in the APC gene (pI1307K), which is associated with increased risk for colorectal cancer (38); and (i) 2 frameshift mutations in *JAK1* (p.K142Rfs*26 and p.L431Vfs*22), which are commonly observed in microsatellite instability-high tumors (39). Notably, despite *ERF* copy loss, abundant ERF transcripts were measured by RNA-Seq in LNCaP_FGC cells, and high outlier levels of RAF1 transcripts were not detected (Supplemental Figure 1D).

We characterized the context of the mutations to infer the mutational processes underlying the damaging event (see Methods). The predominant type of SNV involved G-to-A and C-to-T transitions. Mutational signature analysis using SigProfiler (40) determined that LNCaP_FGC mutations were attributed to 7 single base substitution (SBS) signatures: SBS5 (aging/clock-like), SBS8 (unknown/HR deficiency), SBS12 (unknown), SBS14 (polymerase epsilon mutation and defective mismatch repair [MMR]), SBS21/SBS44 (defective MMR), and SBS30 (base excision repair deficiency) (Supplemental Figure 1, E and F, and Supplemental Table 3) (41). These data support a hypermutation process driven by DNA mismatch-repair deficiency, which is consistent with the observed biallelic structural loss of *MSH2/MSH6*.

We compared the LNCaP_FGC genomic features generated herein, with 2 previous WGS assessments of the LNCaP genome, hereafter LNCaP_SRR7943697 and LNCaP_FGC_PRJNA361316 (23, 42), and confirmed the major oncogenic events identified in our analysis, including the *AR* T878A mutation, *ETV1* gene rearrangement, biallelic *PTEN* loss, and *MSH2/6* biallelic copy loss (Figure 1E and Supplemental Table 2). The overall numbers of SNVs, insertions/deletions, and structural genomic alterations approximated our findings, confirming a hypermutated genome with underlying mismatch DNA repair deficiency and microsatellite instability (Supplemental Table 1). However, the LNCaP_SRR7943697 genome diverged substantially from both the LNCaP_FGC genome derived here and LNCaP_FGC_PRJNA361316, with a higher number of SNVs that were in common with other LNCaP strains such as LNCaP_C4 (Supplemental Figure 2A). Notably, cells for the LNCaP_SRR7943697 analysis were provided by the laboratory of Leland Chung, University of Virginia, Charlottesville, Virginia, USA, who developed the LNCaP_C4 strains, supporting a common origin (personal communication, Bryce Paschal and Daniel Goeili, University of Virginia). A specific comparison of the LNCaP_FGC genome derived here and LNCaP_FGC_PRJNA361316 determined that the vast majority of SNVs and insertions/deletions (3942/4680, 84.2%) and structural alterations (261/321, 81.3%) were shared between these isolates, but numerous events were also private to each genome (Supplemental Figure 1, G and H, and Supplemental Table 1).

A presumed pathogenic subclonal SNV (NM_000546.5:c.700T>C: p.Y234H) in *TP53* exon 7 was identified in LNCaP_SRR7943697, but we did not observe this variant by WGS in the LNCaP_FGC isolate used in the present study, nor was it reported in the LNCaP_FGC_PRJNA361316 WGS analyses (42). A prior whole-exome sequencing (WES) analysis of LNCaP_FGC did not report the Y234H mutation, but identified a P72R *TP53* variant, which likely represents a common germline polymorphism. We confirmed P72R in each of the LNCaP_FGC WGS assessments (43). To further investigate the discrepancy involving the Y234H variant, we evaluated the *TP53* locus in our LNCaP_FGC cells using a capture-based sequencing platform, UW-OncoPlex, designed to assess the mutation and copy-number status of 400 cancer-associated genes (44). At an average read-depth of 700× across the *TP53* exons and 1,300 reads at this specific nucleotide, 2 reads corresponded to the *TP53* c.700T>C:p.Y234H variant (0.15%). However, as noted below, this mutation was observed at higher frequencies in other LNCaP-derived substrains, providing evidence that LNCaP_FGC comprises a heterogenous population of cells (7, 43, 45).

### Comparative assessments of LNCaP-derived cell strain genomes.

**Figure 2:**
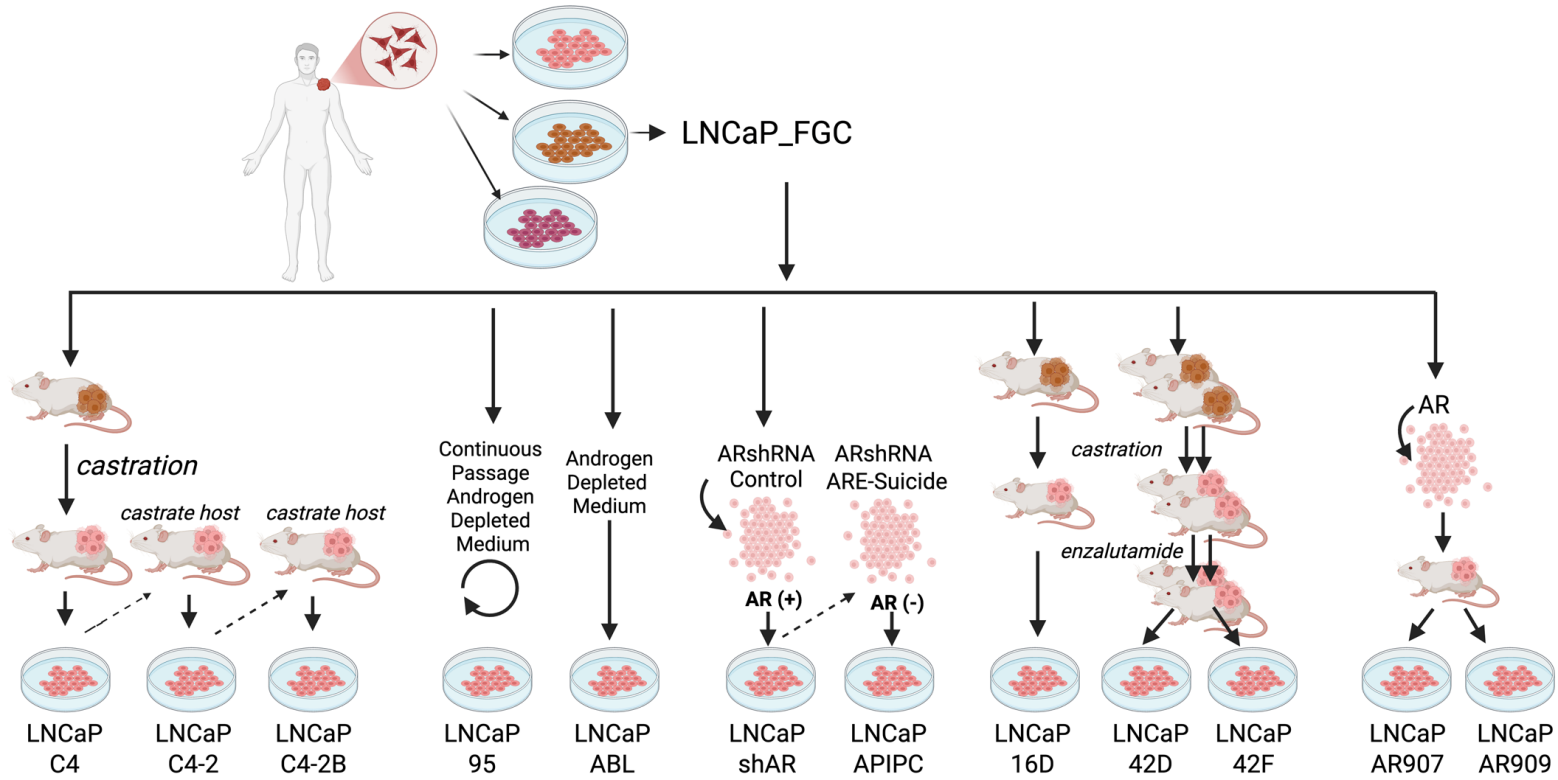
Relationships and approaches used to generate LNCaP_FGC and associated strains.

Cell strains derived from LNCaP_FGC exhibit a range of phenotypes that include resistance to AR-targeting therapies and the acquisition of metastatic capabilities in vivo (10, 14, 17). We compared the genomes of LNCaP_FGC and 12 derivative strains to identify genomic alterations that potentially underlie the diverse phenotypes of these models (Figure 2). In addition to LNCaP_FGC, we analyzed in vivo–derived castration-resistant strains LNCaP_C4, LNCaP_C4-2, LNCaP_C4-2B (10), and LNCaP_16D (14); in vivo–derived enzalutamide-resistant strains LNCaP_42D and LNCaP_42F (14); in vitro–derived androgen deprivation therapy-resistant LNCaP_ABL and LNCaP_95 strains resulting from continuous in vitro passage in androgen-depleted medium (15, 17); the AR-null LNCaP_APIPC strain and its parental strain LNCaP_shAR (21); and two strains with overexpression of the WT AR, LNCaP_AR907 and LNCaP_AR909 (13, 46, 47). Each strain was propagated under growth conditions reported in the studies that detailed their derivation (see Methods).

**Figure 3:**
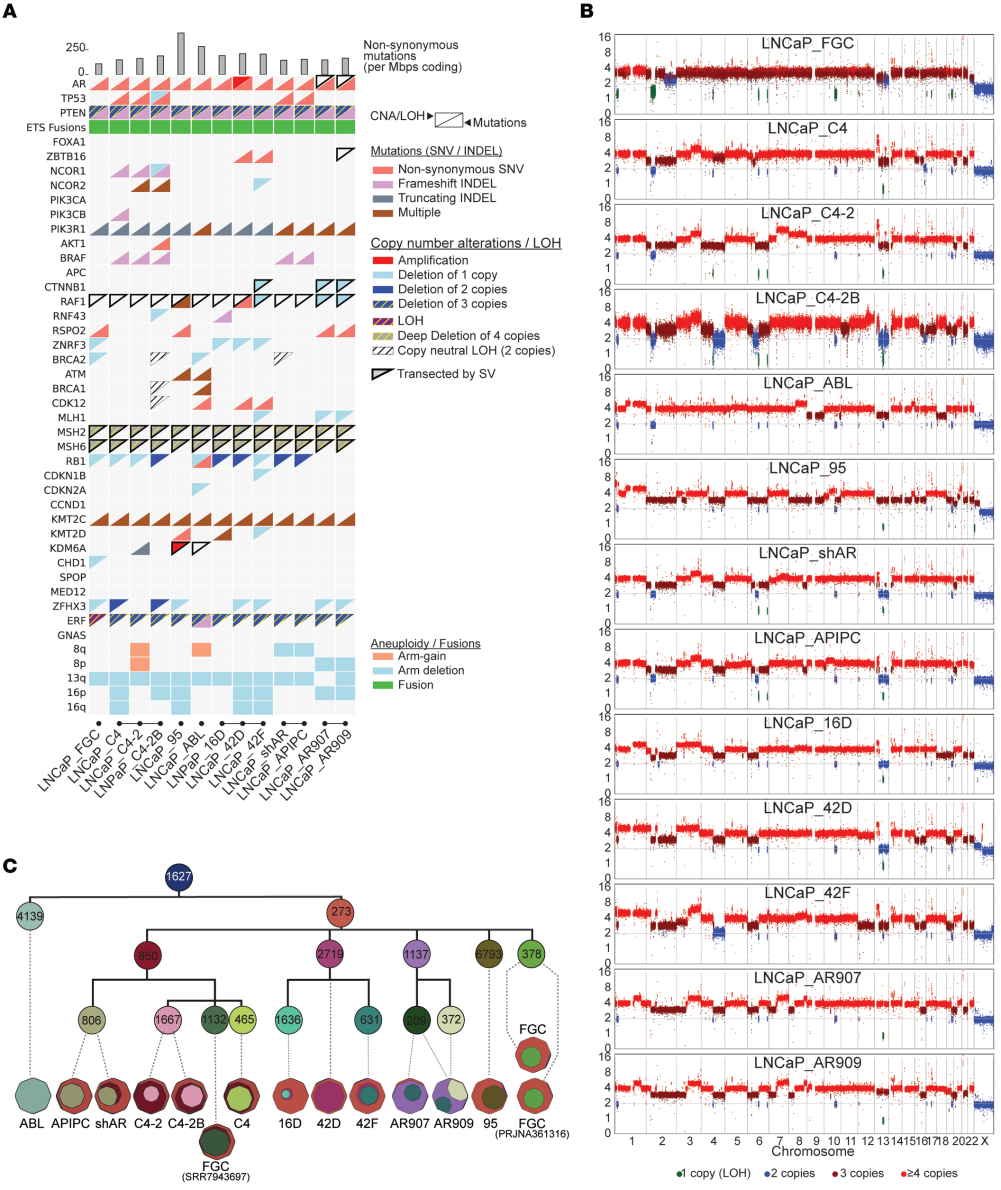
LNCaP substrains exhibit recurrent and unique genomic alterations. (**A**) Mutation and copy number alteration (CNA) status for selected genes with recurrent alterations in metastatic prostate cancer across LNCaP strains. The bottom right triangle indicates an observed pathogenic mutation (SNV/INDEL) within the gene, and the top left triangle indicates CNA status (amplification, shallow deletions, deletions, deep deletions) that overlap the gene. CNA events that also have loss of heterozygosity (LOH) status are depicted with a hatch pattern. “Multiple” indicates instances where 2+ pathogenic mutations are observed for that gene. Tumor mutation burden was computed as the number of nonsynonymous mutations per megabase pairs of coding regions (top). Aneuploidy status (arm gain, arm deletion) is indicated for select chromosome arms. Fusion status indicates evidence for genomic rearrangement involving an ETS transcription factor. Genes that have been transected by at least 1 of 2 breakpoints of a structural variation (SV) event are indicated with a black border around the triangle. (**B**) Genome-wide integer copy number profiles generated by TitanCNA. Data points represent individual germline heterozygous SNPs or 10 kb pair-sized bins. Estimated integer copy number (*y* axis) is indicated by colors: deletions (green), copy neutral (blue), gain (dark red), and amplifications (bright red). (**C**) Cell-lineage tree reconstruction based on inferred subclonal composition using all unique pathogenic SNV mutations (24,282) across the substrains. SNVs included in the analysis had filtering criteria of presence in COSMIC Gene Census; deleterious status by SIFT, LRT, MutationTaster, FATHMM, and ClinVar; 1% or less in gnomAD or ExAC databases; and 10 or more total reads and 3 or more mutant reads in the tumor. PyClone-VI was used to determine cellular prevalence and clonal clusters; LICHeE for lineage reconstruction; cloneMap for visualization. INDEL, insertions/deletions; SNV, single nucleotide variant.

The majority of prostate carcinoma–associated genomic alterations identified in LNCaP_FGC were concordantly identified in all derived strains (Figure 3, A and B, Supplemental Figure 2B, and Supplemental Tables 2 and 3). The estimated tumor ploidy of the strains ranged from 3.24 to 3.91, and the majority of copy number and structural alterations identified in LNCaP_FGC were retained in the substrains, though each harbored additional unique chromosomal losses, gains, and rearrangements (Supplemental Figure 2, C and D, and Supplemental Tables 2 and 3). For example, relative to LNCaP_FGC, the LNCaP_C4-2B genome comprised 7 additional regions of large copy loss, 6 regions of copy gain, and 123 gene rearrangements (Supplemental Table 3). Of interest, the LNCaP_C4-2B copy number profile was more similar to LNCaP_16D and LNCaP_42D (Pearson’s *r* > 0.63) compared with LNCaP_FGC (*r* = 0.30) and LNCaP_C4-2 (*r* = 0.58) by correlation analysis (Supplemental Figure 2D). We also confirmed an 8q24 amplification in LNCaP_ABL with a copy gain that included the MYC enhancer (48) (Supplemental Tables 2 and 3). We determined that LNCaP_42D harbors an amplification of the AR and AR enhancer, which is unique among all of the strains evaluated, and may reflect the selective pressure of exposure to enzalutamide and subsequent resistance (Supplemental Figure 2E).

Analyses of the LNCaP_FGC genome demonstrated 2 copies of the Y chromosome, and this result is concordant with previous cytogenetic assays of the original LNCaP line, demonstrating a high degree of aneuploidy with chromosome numbers ranging from 33 to 91 and the presence of both X and Y chromosomes (7, 22). However, sequence reads mapping to the Y chromosome were markedly diminished or absent from the genomes of LNCaP_95, LNCaP_42D, and LNCaP_42F, indicating loss of the Y chromosome in these strains (Supplemental Figure 2F and Supplemental Table 3). A previous study evaluating the contribution of the Y-encoded gene *KDM5D* demonstrated that parental LNCaP_FGC cells comprise a mixed population with approximately 90% of cells harboring 2 Y chromosomes and approximately 10% with 1 Y chromosome, whereas the LNCaP_C4-2 strain comprises approximately 5% of cells with 2 copies, 30% with 1 copy, and approximately 60% with complete Y chromosome loss (49). These results were extended by analyzing the expression of Y-encoded genes by RNA-Seq (see below, *The LNCaP_FGC transcriptome)*.

The number of SNVs across LNCaP substrains ranged from 382,588 to 1,503,051, demonstrating that all strains were hypermutated with more than 10 SNVs/Mbp (Supplemental Table 2). These included SNVs that were specific to particular strains, including a large fraction not identified in LNCaP_FGC cells as well as SNVs present in LNCaP_FGC that were not present in particular substrains (Supplemental Figure 2G). We analyzed the lineage relationships of the strains by comparing the presence of coding SNVs and insertions/deletions, rooting a phylogenetic tree to the LNCaP_FGC line we obtained from ATCC (Supplemental Figure 2A). The published LNCaP_FGC_PRJNA361316 WGS was the nearest branch point based on 1,223 coding mutations, whereas LNCaP_95 diverged to the greatest extent, with 14,726 gained coding mutations, equaling 5 mutations/MB genome-wide. Genomic clonality analysis and construction of the clonal lineage tree revealed unique subclonal mutations (range 209 to 6,793) exclusive to individual substrains, confirming divergent clonal relationships (Figure 3C and Supplemental Table 3). Together, these results highlight sustained mutagenesis in substrains during their derivation and propagation, with the continued accumulation of a large number of mutations over years of continuous passage in culture.

The LNCaP-derived strains retained a similar composition of mutational signature profiles compared with LNCaP_FGC, but with increased numbers of mutations comprising each signature with the predominant gains in SNVs corresponding to mutations conferred by mismatch repair deficiency (MMRd) (Supplemental Figure 2H). Additional signatures of defective DNA repair (SBS21/SBS26) were also detected in LNCaP_42D, LNCaP_42F, and LNCaP_95. A limitation for these analyses is the lack of a normal germline reference for LNCaP_FGC. When we used LNCaP_FGC as the reference for determining differential mutations in substrains, the majority of gained mutations were classified as aging/clock-like (SBS5) and MMRd (SBS14 and SBS44) (Supplemental Figure 2I).

The ongoing MMR deficiency and cellular heterogeneity of LNCaP challenges accurate assessments of whether new mutations represent driver versus passenger events, even for genes with bone fide effects in promoting neoplasia. As noted above, a previous study of the LNCaP_FGC genome reported a deleterious SNV in *TP53* (c.700T>C:p.Y234H) (23). We observed this event at a very low frequency in LNCaP_FGC, where a capture-based method with 1000× coverage identified this SNV at 0.15% variant allele fraction. In most substrains, the allele fraction of this *TP53* SNV remained at the level of LNCaP_FGC or was not detected at all, whereas in several substrains, LNCaP_C4, LNCaP_C4-2, LNCaP_C4-2B, and LNCaP_APIPC, this mutation was evident at substantially higher variant frequencies, ranging from 23% to 33% of reads. Other notable genomic differences between strains included biallelic mutations in *POLE* in LNCaP_95, predicted pathogenic mutations in *ATM* and *RB1* in LNCaP_ABL, a subclonal mutation in *FANCA* in LNCaP_C4-2 (but not LNCaP_C4), a subclonal FOXA1 p.E292* stop-gain mutation in LNCaP_C4-2, and subclonal mutations in *KMT2D* in LNCaP_16D (but not LNCaP_42D or 42F) (Supplemental Table 3).

Although the genomes of the LNCaP substrains diverge in interesting and important ways with respect to known drivers of neoplasia, metastasis, and treatment resistance in human prostate carcinoma, the underlying genomic structure and nucleotide sequence are largely similar. This has important implications for distinguishing substrains for cell line verification because the short-tandem repeat (STR) profiles commonly used to differentiate cell lines do not discriminate between LNCaP_FGC and the common substrains evaluated here. To address this, we assembled a panel of SNVs that are unique to each strain and refined the list to focus on SNVs in expressed genes, such that strains can be distinguished by RNA-Seq as well as WES and WGS (Supplemental Table 3). However, the heterogeneity and instability of LNCaP indicates that prolonged culture may result in genomes that diverge from this reference set.

### The LNCaP_FGC transcriptome.

**Figure 4:**
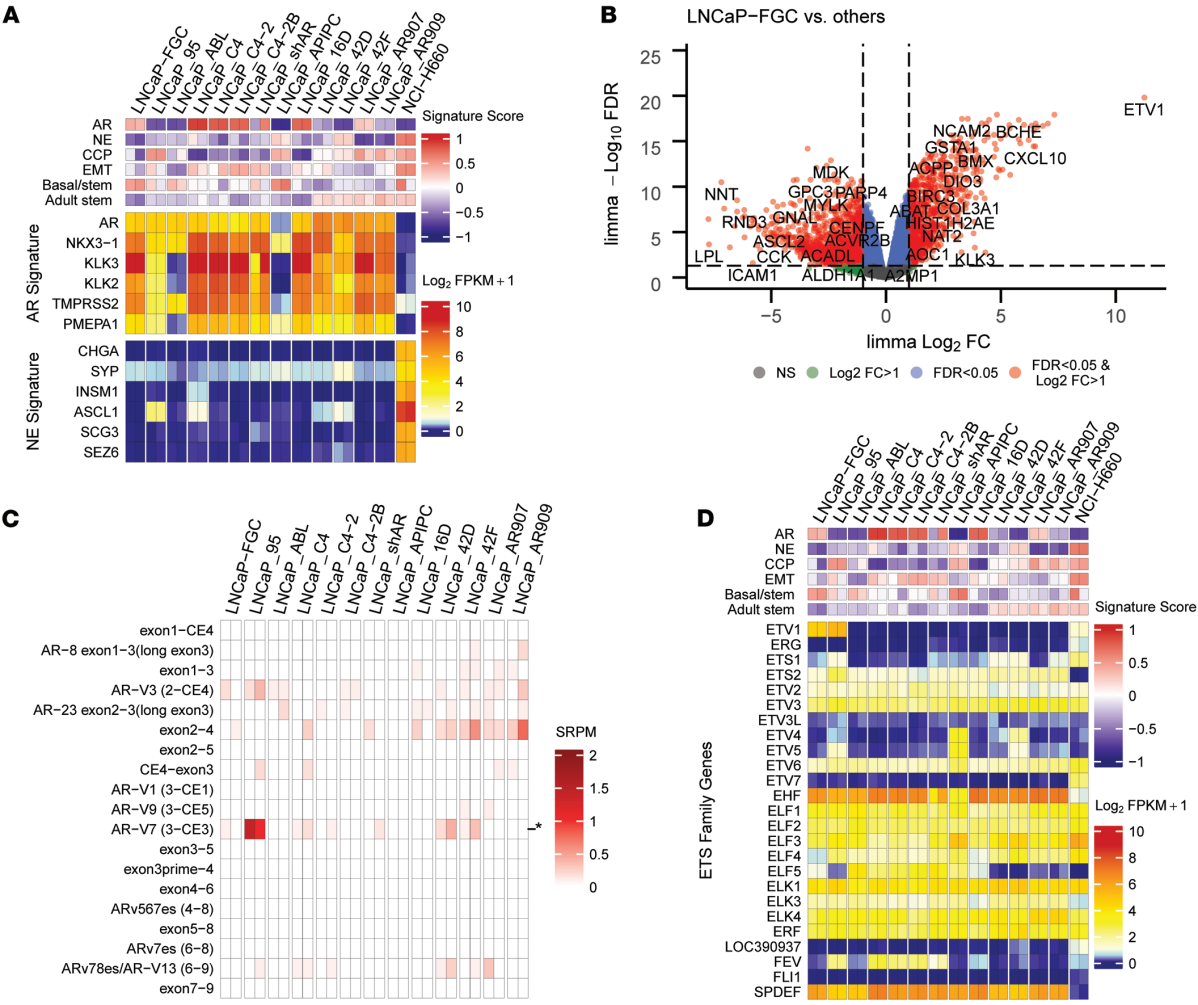
Genes differentially expressed between LNCaP substrains include AR splice variants and ETS transcription factors. (**A**) Heatmap of transcript abundances of selected AR activity and NE phenotype genes and signature scores. Gene set variation analysis (GSVA) signature scores and log_2_ FPKM values are colored according to scales shown on plot. AR, androgen receptor; NE neuroendocrine; CCP, cell cycle progression; EMT, epithelial-mesenchymal transition. (**B**) Representative volcano plot of differential expression analysis between LNCaP_FGC and 12 LNCaP substrains. (**C**) Heatmap of AR splice variant expression across LNCaP strains by spliced reads per million (SRPM) color scale. AR-V7 expression is indicated with an asterisk. (**D**) Heatmap of transcript abundances of selected ETS family genes and signature scores. GSVA signature scores and log_2_ FPKM values are colored according to scales shown on plot. FPKM, fragment per kilobase per million mapped reads.

To evaluate the repertoire of genes expressed in the LNCaP_FGC line, we used whole-transcriptome RNA-Seq of polyA-selected mRNA extracted from biological replicate cultures. A threshold of 1 fragment per kilobase per million mapped reads (FPKM) classified a gene as expressed. Using this metric, the LNCaP_FGC line, grown under standard culture conditions of RPMI with 10% FBS, expressed 10,808 genes (≥1 FPKM in both replicates). The LNCaP_FGC transcriptome is notable for AR expression (at 24 FPKM), ranked as the 1,948th most abundant transcript, and for a spectrum of genes previously reported to be transcriptionally regulated by AR activity, including *NKX3-1*, *KLK2*, *KLK3*, and *TMPRSS2* (Figure 4, A and B) (50, 51). The LNCaP_FGC line does not express transcription factors associated with neuroendocrine phenotypes, such as *ASCL1* or *NEUROD1*, or transcripts encoding neuroendocrine-associated proteins, including CGA or SYP when grown in standard steroid-replete growth medium (Figure 4A). The top 30 most abundant transcripts in LNCaP_FGC primarily encode ribosomal proteins, though *KLK3*/PSA was the 28th most abundant transcript (Supplemental Figure 3, A and B).

AR splice variants that encode an AR protein lacking the ligand binding domain have emerged as potential drivers of prostate carcinoma resistance to AR-directed therapy (52–54). LNCaP_FGC expressed detectable but very low levels of AR splice variants corresponding to AR-V3 (0.172 spliced reads per million [SRPM]) and AR-V7 (0.086 SRPM) (Figure 4C). As noted above, LNCaP_FGC cells harbor a structural genomic alteration involving the ETS family member *ETV1*, which comprises a complex rearrangement involving *MIPOL1* and *DGKB* loci, resulting in high ETV1 expression (ETV1 FPKM = 24) (26) (Figure 4, B and D). The expression of other ETS family members ranged from 0 to 79 FPKM, with highest expression of EHF and SPDEF (Figure 4D). Several other expressed gene fusions were identified in LNCaP_FGC with evidence for underlying structural alterations, including *RERE-PIK3CD* and *MRPS10-HPR* (Supplemental Figure 3C and Supplemental Table 4, E and F).

**Figure 5:**
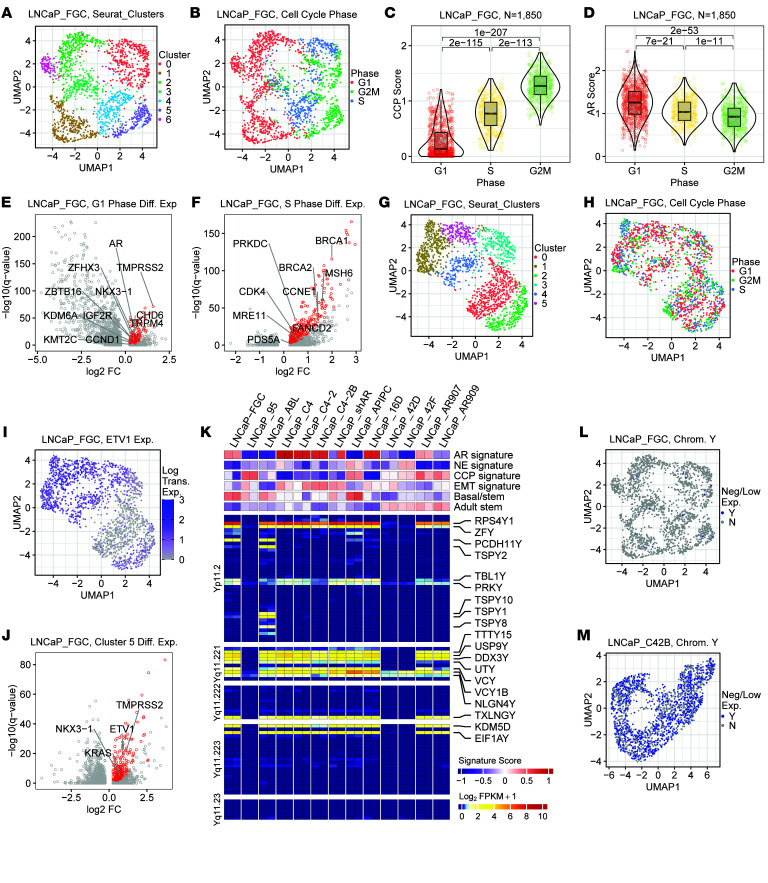
Y chromosome and transcriptional heterogeneity within the LNCaP_FGC line. (**A**) Single-cell RNA-Seq (sc-RNA-Seq) UMAP of LNCaP_FGC cells where colors represent clusters identified using Seurat FindClusters with resolution set to 0.5. (**B**) scRNA-Seq UMAP of LNCaP_FGC where colors represent clusters defined by cell cycle phase: G1 (*n* = 883 cells), G2/M (*n* = 513cells), and S (*n* = 454 cells). (**C**) Cell cycle progression (CCP) signature score in LNCaP cells partitioned by cell cycle. Wilcoxon *P* values show significant differences between cells in phases of the cell cycle. (**D**) Androgen receptor (AR) activity score in LNCaP_FGC cells partitioned by cell cycle. AR scores quantified per cell by the average log-transformed count of AR signature genes with median counts greater than 0. Wilcoxon *P* values shown. (**E** and **F**) Volcano plot demonstrating genes differentially expressed in LNCaP_FGC cells in G1 (**E**) or S (**F**) versus cells in different phases of the cell cycle. Genes denoted by red color are significantly differentially expressed (log_2_ fold change > 0, *q* value < 0.05, G1 expressed > 50%). (**G** and **H**) sc-RNA-Seq UMAP of LNCaP_FGC cells following regression of cell cycle–associated effects with cells annotated by cycle phase (**H**). (**I**) sc-RNA-Seq UMAP of LNCaP_FGC highlighting ETV1 expression; 33% of cells lacked ETV1. (**J**) Volcano plot demonstrating genes differentially expressed in LNCaP_FGC cells assigned to cluster 5 versus other clusters from **G**. (**K**) Heatmap of transcript abundance of genes on the Y chromosome and signature scores. Genes with detectable expression in at least 1 sample are listed on the right side of the plot. (**L** and **M**) UMAP of (**L**) LNCaP_FGC and (**M**) LNCaP_C4-2B, where cells colored blue have negligible expression of all Y chromosome genes (≤ 1 read mapping to any Y chromosome gene, with a maximum of 5 total reads mapping to Y chromosome genes). Data were downsampled to be comparable (1,850 cells, 19,000 average reads). UMAP, uniform manifold approximation and projection.

To further assess the heterogeneity of the LNCaP_FGC line that may not be apparent in bulk RNA-Seq, we performed single-cell RNA-Seq (scRNA-Seq) to delineate the gene expression repertoire of individual cells. This analysis revealed several subclusters of LNCaP_FGC cells, with a major influence denoted by cell cycle–related gene expression (Figure 5, A and B). In steady-state growth conditions, 48%, 24%, and 28% of LNCaP_FGC cells partitioned into G1, S, and G2M cell cycle phases, in which G2M cells express significantly higher levels of cell cycle progression signature genes (Figure 5C). Notably, AR-regulated genes were expressed most highly in G1 cells (Figure 5, D and E), whereas genes involved in DNA repair processes were expressed in cells in the S phase (Figure 5F). When cell cycle gene expression was regressed out of the analysis, distinct clusters remained evident and G1, G2M, and S phase cells were distributed across clusters (Figure 5, G and H). Cells partitioned to cluster 0 lacked ETV1 expression (Figure 5I), comprising approximately 33% of the LNCaP_FGC population, a finding relevant to the studies of LNCaP substrains described below. Cluster 5 cells expressed the highest levels of ETV1 and AR-regulated genes (Figure 5, G and J). The expression of genes encoded on the Y chromosome, such as *DDX3Y*, *EIF1AY*, *RPS4Y1*, and *USP9Y*, was not detected in 0.7% of LNCaP_FGC cells (Figure 5, K and L), supporting prior studies indicating Y chromosome loss in a small percentage of LNCaP_FGC by FISH analysis (49).

### Comparative assessments of LNCaP substrain transcriptomes.

**Figure 6:**
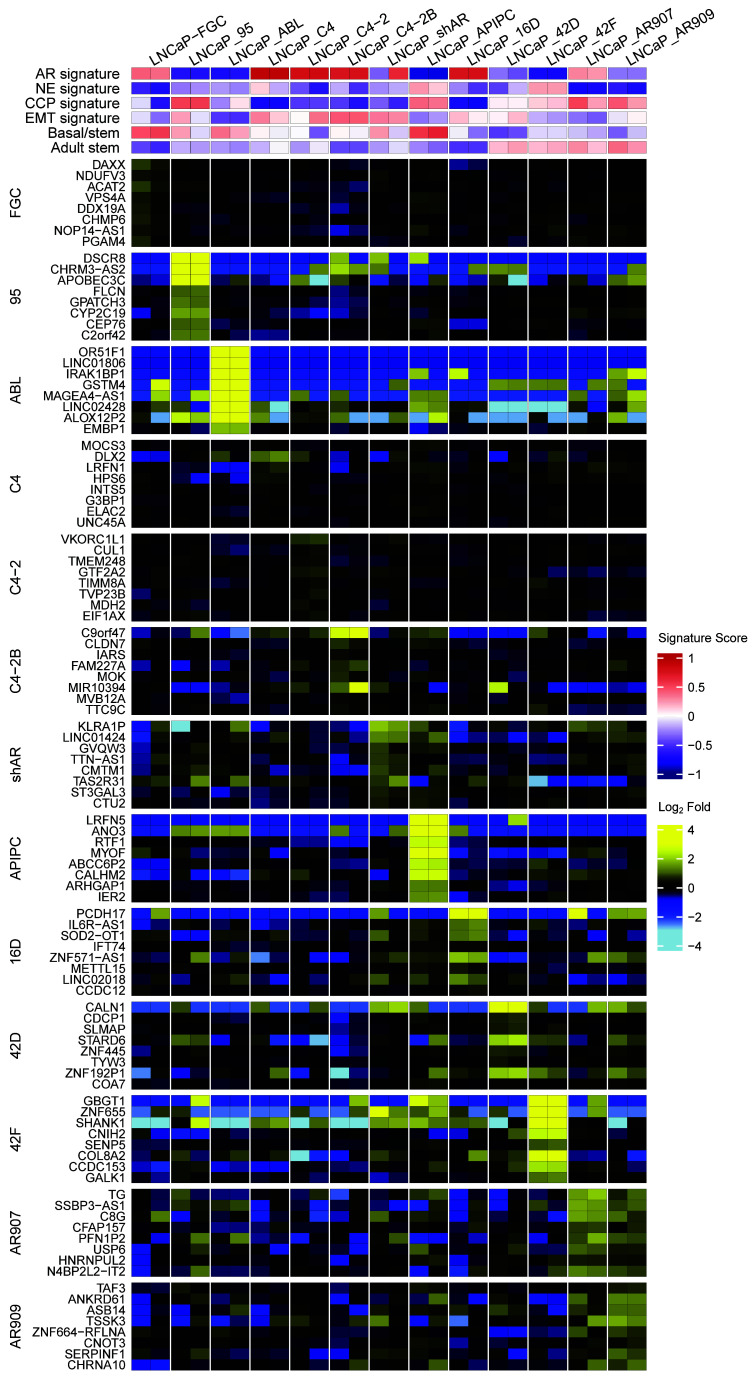
LNCaP substrains express divergent transcriptomes. Heatmap of the top 8 genes upregulated uniquely in each strain compared with the 12 other strains, with FDR less than 0.05. Gene set variation analysis scores and log2 relative fold ratios are colored according to scales shown on the plot.

LNCaP substrains have been developed with the objectives of understanding mechanisms driving resistance to AR inhibition and processes promoting metastatic potential (10, 14, 17, 18, 21, 55). We systematically compared the transcriptomes of LNCaP_FGC and 12 strains by pairwise comparison of each strain against the other 12 (Figure 4, A and B, Figure 6, and Supplemental Table 4). The composite transcriptomes combining all LNCaP substrains comprised 13,574 genes out of a potential 27,363 genes encompassing the human transcriptome. Overall, 8,932 genes were expressed in all strains. Compared to all other strains, 439 genes were uniquely increased in LNCaP_FGC, and 790 genes were differentially decreased (FDR < 0.05; fold change, 3-fold; Figure 4B and Supplemental Figure 3D). For every pairwise comparison, hundreds of genes were differentially expressed using a threshold of fold change greater than 3 and FDR less than 0.05 (Supplemental Figure 3D, Supplemental Figure 4, and Supplemental Table 4). Every LNCaP substrain expressed subsets of genes uniquely, though the extent varied by the lineage relationships (Figure 6 and Supplemental Figure 4A); for example, the transcriptomes of LNCaP_C4 and LNCaP_C4-2 cells were nearly superimposable, whereas LNCaP_16D differed substantially from LNCaP_AR909. Notably, lineage relationships established by shared mutations were maintained when evaluating the similarity of LNCaP substrains by gene expression where relationships were aligned by exposure to androgens, AR antagonists, and AR activity (Supplemental Figure 5A).

Alterations in the *AR* are commonly observed to occur in castration-resistant prostate carcinoma and documented to play causal roles in driving resistance to ADT and androgen receptor signaling inhibitor therapy (56). AR events include LBD mutations, the expression of AR splice variants, *AR* copy gain, *AR* enhancer amplification, and the inclusion of *AR* in extra-chromosomal DNA (56–58). Other resistance mechanisms to ADT/ARSI involve phenotype transitions to lineages that lack AR expression or activity (20, 21, 59). Several of the LNCaP_FGC substrains were developed after resistance to ADT, such as LNCaP_C4-2B, LNCaP_95, LNCaP_ABL, and LNCaP_16D, or after ARSI treatment, such as LNCaP_42D and LNCaP_42F. All of the LNCaP substrains, except LNCaP_APIPC, which was engineered to completely eliminate AR activity, expressed the AR, the AR-regulated homeobox gene NKX3-1, and subsets of known AR targets with variation associated with the culture conditions for strains that reflect growth in androgen-replete versus depleted medium (Figure 4A). Assessments of the *AR* locus identified no structural alterations such as AR amplification, enhancer copy gain, or rearrangement in any strain, including those resistant to ADT or enzalutamide, except LNCaP_42D, which we found to harbor an amplification of the *AR* locus (Supplemental Figure 2E). Of interest, only LNCaP_95 cells expressed high levels of AR splice variant 7 (AR-V7; average SRPM of 2 replicates = 1.2), despite other strains also demonstrating resistance to androgen deprivation or ARSI exposure (Figure 4C). A mechanism driving AR-V7 expression predominantly in LNCaP_95 remains to be established.

Androgen deprivation has been reported to promote a neuroendocrine-like phenotype in LNCaP_FGC cells, with the induction of neuroendocrine-associated genes such as SYP after androgen withdrawal (60, 61). Of interest, LNCaP substrains adapted to proliferate in androgen-depleted medium or under ARSI treatment did not activate a full neuroendocrine program, though LNCaP_95, LNCaP_42D, and LNCaP_42F cells expressed modestly higher levels of the neural transcription factor *ASCL1*, and SYP expression was increased in LNCaP_42F (Figure 4A). Although these differences are notable relative to LNCaP_FGC cells, which completely lack expression of these transcripts, they did not approach the levels measured in the small-cell NEPC LuCaP49_CL model or the NCI_H660 NEPC line, where *ASCL1* and *SYP* were 188-fold and 8-fold greater than measured in LNCaP_42F cells (Supplemental Figure 3, E and F) (62). Further, despite the low-level induction of transcripts associated with neuroendocrine differentiation, each LNCaP strain maintained high levels of AR expression and evidence of continued AR program activity (Figure 4A).

As detailed above, LNCaP_FGC cells harbor a complex genomic rearrangement that includes a *MIPOL1-DGKB* interchromosomal gene fusion accompanied by the cryptic insertion of *ETV1* from chromosome 7 into *MIPOL* on chromosome 14 (26). RNA-Seq analysis confirmed that LNCaP_FGC cells express high levels of ETV1 transcripts (24 FPKM). Of interest, although the *MIPOL1-DGKB* fusion event is evident in all of the other LNCaP substrain genomes, only LNCaP_95 also expresses ETV1 (Figure 4D). ATAC-Seq analysis of the *ETV1* locus across the LNCaP substrain did not reveal a pattern that explained differential *ETV1* expression across the strains (Supplemental Figure 5B). Although other ETS family members such as ERG comprise recurrent oncogenic gene rearrangements in prostate carcinoma, we did not identify a pattern of expression of other ETS genes that could substitute for the loss of ETV1 function across LNCaP strains (Figure 4D).

Analyses of the LNCaP substrain genomes identified variability in the presence of DNA reads mapping the Y chromosome (Supplemental Figure 2F). Bulk RNA-Seq confirmed that LNCaP_95, LNCaP_42D, and LNCaP_42F lacked transcripts from genes encoded on the Y chromosome including *KDM5D*, *UTY*, and *EIF1AY* (Figure 5K). Single-cell analysis of LNCaP_FGC and LNCaP_C42B identified 0.7% and 60% of cells, respectively, lacking expression (≤1 read per gene) of Y chromosome genes (Figure 5, L and M). Although a report using FISH probes to quantitate cells with Y chromosome loss identified 30% and 60% of LNCaP_C4 cells with single or complete Y chromosome loss, we did not observe lower levels of Y-encoded transcripts in this strain relative to LNCaP_FGC (Figure 5K) (49). This discrepancy prompted an analysis of publicly available RNA-Seq data from LNCaP substrains. Across multiple studies, LNCaP_FGC consistently retains Y chromosome gene expression. No other RNA-Seq data were available for LNCaP_C4. Of interest, LNCaP_ABL and LNCaP_C4-2B were more variable, with a subset of samples lacking evidence of Y chromosome gene expression (Supplemental Figure 5C and Supplemental Table 4).

### Variation in LNCaP chromatin landscapes across LNCaP substrains.

**Figure 7:**
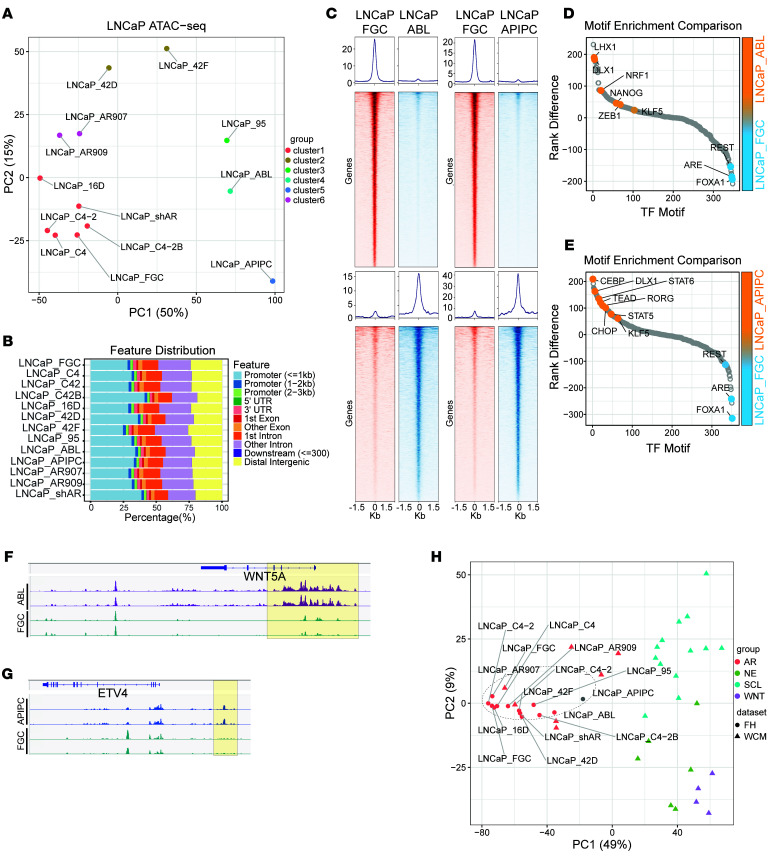
Chromatin profiles associate with LNCaP substrains that exhibit resistance to ADT and ARSI exposure. (**A**) Unsupervised PCA using the top 5,000 most variable accessible peaks across the LNCaP substrains. Consensus hierarchical clustering identified 6 distinct groups or clusters on LNCaP strains. (**B**) ATAC-Seq peak annotation distribution of the mapped ATAC-Seq peaks across LNCaP substrains. (**C**) Heatmap representing ATAC-Seq signal intensity at specific genomic loci in LNCaP_FGC, LNCaP_ABL, and LNCaP_APIPC cells. (**D** and **E**) Differential transcription factor binding motif enrichment within a 250-bp window surrounding ATAC-Seq peaks in LNCaP_FGC, LNCaP_ABL, and LNCaP_APIPC strains. (**F** and **G**) ATAC read density differentially mapped to the *WNT5A* and *ETV4* loci in LNCaP_FGC, LNCaP_ABL, and LNCaP_APIPC strains. (**H**) Unsupervised PCA plot of the top 5,000 most variable accessible peaks across LNCaP substrains integrated with cell lines and organoid ATAC assessments from Tang et al. (64) partitioning tumors into AR, neuroendocrine (NE), stem cell-like (SCL), and WNT subtypes. Dotted circle encompasses all LNCaP strains.

To develop an understanding of the gene regulatory landscape that may underlie LNCaP phenotypes, we performed assays for transposase-accessible chromatin sequencing (ATAC-Seq) in LNCaP_FGC and each of the 12 substrains. For LNCaP_FGC, we identified 45,323 reproducible peaks. Top-ranked sequence motifs enriched in these peaks included the palindromic AR response elements and FOXA1 sites. Comparative assessments of the regions showing the most variable accessibility using consensus hierarchical clustering identified 6 substrain groups (Figure 7A). Genomic regions with differential accessibility associating with each group, defined as those with log_2_(fold change) greater than 3 and *q* value less than 0.01, were enriched at enhancers relative to promoters (*P* = 4.8 × 10**^–14^**, OR = 1.7), as observed in a prior study of chromatin-accessible regions that associate with distinct castration-resistant prostate cancer phenotypes (Figure 7B) (63, 64).

Previous studies identified notable differences in chromatin accessibility in enzalutamide-resistant LNCaP_42D compared with enzalutamide-sensitive LNCaP_16D cells with the enrichment of motifs for ASCL1, GATA, and NANOG transcription factor binding (19). The AR-null LNCaP_APIPC and androgen-repressed LNCaP_ABL strains also had markedly distinct ATAC-Seq profiles: 19,747 and 14,850 differential peaks (log_2_ fold change ≥2 and *q* value ≤ 0.05) compared with LNCaP_FGC (Figure 7C). Motifs enriched in LNCaP_FGC relative to LNCaP_ABL and LNCaP_APIPC included FOXA1, REST, and AR response elements, whereas motifs enriched in LNCaP_ABL included LHX1, DLX1, NRF1, and KLF5, and those in LNCaP_APIPC included CEBP, STAT, and KLF5 (Figure 7, D and E). Overall, both LNCaP_ABL and LNCaP_APIPC exhibited alterations in chromatin accessibility, indicating loss of luminal epithelial identity and gain of neural, basal, and stem-like chromatin organization (65–67). In this context, AR and KLF5 have been reported to drive opposing transcriptional programs, with KLF5 promoting a basal cell–like phenotype and cell migration (68). Analyses of differential transcriptional programs between these strains identified several members of the WNT signaling pathway upregulated in LNCaP_ABL and LNCaP_APIPC, for example, WNT5A and ETV4 (69–71), with corresponding differential ATAC-Seq peaks found at the genomic loci (Figure 7, F and G).

We next sought to determine whether the ATAC-Seq profiles of LNCaP substrains could recapitulate distinct chromatin-based classifications observed in CRPC that comprised AR, neuroendocrine, WNT, and stem cell-like (SCL) categories determined by Tang et al. using prostate carcinoma cell lines and organoid models (64). Specific transcription factors were associated with each of the 4 phenotype groups including AR and FOXA1 for CRPC-AR; NEUROD1 and ASCL1 for CRPC-neuroendocrine; TCF7L2/TCF4 and LEF1/LEF for CRPC-WNT; and FOSL1, JUNB, and ATF3 for CRPC-SCL. We evaluated the expression of these phenotype-defining regulators and found that none of the LNCaP strains differentially expressed high levels of these transcription factors, except for ASCL1 being differentially upregulated in LNCaP_95, LNCaP_C4, LNCaP_42D, and LNCaP_42F relative to LNCaP_FGC, and LEF1 being differentially upregulated in LNCaP_APIPC relative to all other models (Supplemental Figure 5D). However, all strains except LNCaP_APIPC retained a phenotype classification of androgen receptor active prostate cancer (ARPC). Reflecting this result, the chromatin profiles of each LNCaP strain maintained a close relationship with the parental LNCaP_FGC line and not with tumors classified as neuroendocrine, WNT, or SCL (Figure 7H). Notably, while LNCaP_APIPC classified as double-negative prostate cancer by transcriptional output, the chromatin structure maintained alignment with ARPC. These results indicate that although the LNCaP strains express diverse transcriptional programs that indicate a degree of plasticity, their underlying epigenetic architecture has not transitioned to adopt a structure associated with an alternate fully differentiated lineage state.

### Variation in AR cistromes across LNCaP strains.

**Figure 8:**
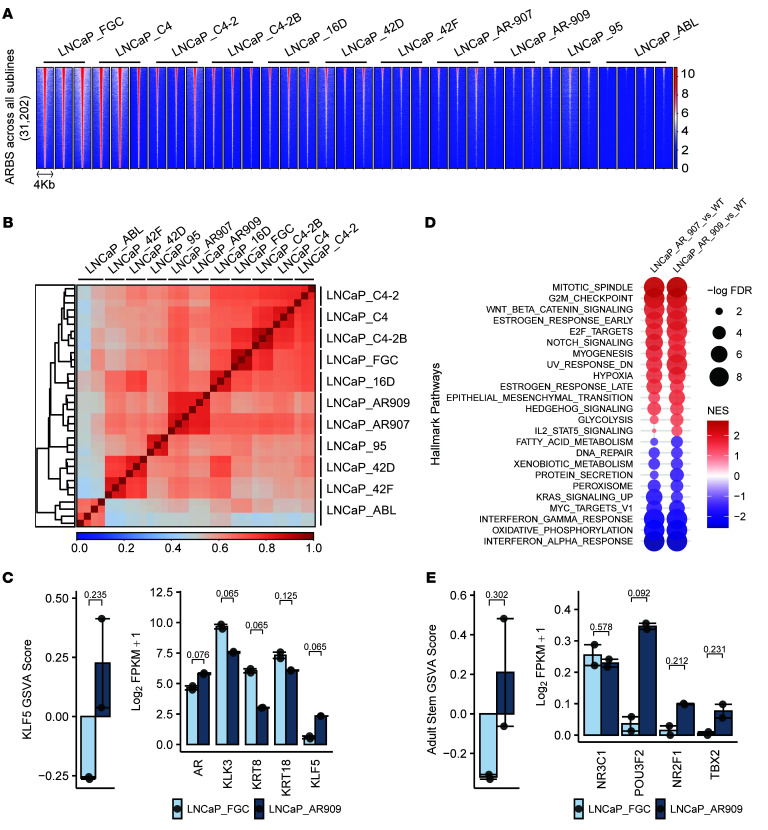
AR cistromes vary across LNCaP substrains. (**A**) ChIP-AR signal across the combined AR binding sites (*n* = 31,202) among all LNCaP strains. (**B**) Spearman’s correlation heatmap of ChIP-AR alignment across all LNCaP strains. (**C**) GSVA scores and transcript abundance by RNA-Seq of KLF5 activity and prostate luminal cell–associated genes. LNCaP_FGC and LNCaP_AR909 (*n* = 2 per line). (**D**) Differentially enriched Hallmark pathways in comparison of LNCaP_FGC to LNCaP_AR907 and LNCaP_AR909 (pathways with FDR less than 0.05 in at least 1 comparison shown.) NES, normalized enrichment score. (**E**) GSVA scores and transcript abundance by RNA-Seq of adult stem cell activity and transcription factors associated with driving ARSI resistance. LNCaP_FGC and LNCaP_AR909 (*n* = 2 per line.)

Activation of the AR drives the expression of several hundred genes comprising the prostate carcinoma transcriptome, which is recapitulated in LNCaP_FGC cells (51). Compared with the transcriptome, the AR cistrome composed of AR binding sites across the genome is more expansive, composed of thousands of binding sites, and has been determined to be altered in the context of prostate neoplasia and castration resistance (72, 73). We next evaluated the AR cistrome under steady-state growth conditions across each of the LNCaP substrains using AR ChIP-Seq. Substantial differences in AR cistromes were observed, which partially reflected the presence or absence of androgens or AR antagonists in the medium (Figure 8, A and B). Notable exceptions were the LNCaP_AR907 and LNCaP_AR909 strains (also known as LNCaP/AR), which are engineered to overexpress the WT AR in the genomic background of AR T878A mutation but retain sensitivity to enzalutamide treatment (18, 46, 47). Although LNCaP_FGC, LNCaP_AR907, and LNCaP_AR909 were all propagated in the same standard FBS medium, approximately 9,900 AR binding sites were identified in LNCaP_FGC, whereas only approximately 1,400 and approximately 500 AR binding sites were identified in LNCaP_AR907 and LNCaP_AR909, respectively (Figure 8A and Supplemental Figure 6, A and B). The addition of 10 nM of the synthetic androgen R1881 produced a full recovery of the AR cistrome, with approximately 32,000 AR binding sites in AR_907 and approximately 24,000 AR binding sites in AR_909, which approximated the roughly 32,000 AR binding sites in LNCaP_FGC after R1881 exposure (Supplemental Figure 6, A and B).

Although AR binding to well-characterized genes involved in the prostate secretory program, such as *KLK2* and *KLK3,* was diminished in LNCaP_AR909, AR binding to genes involved in cell proliferation, such as *MCM7,* was retained, indicating that while overall AR binding was reduced genome-wide, the contribution of AR to cell proliferation was maintained (Supplemental Figure 6, C and D). We confirmed this observation by immunoblot, demonstrating near absence of PSA protein in LNCaP_AR909 grown in steady-state conditions, with a modest increase after the addition of R1881 (Supplemental Figure 6E).

In prior studies, the AR has been shown to be growth repressive when overexpressed in prostate carcinoma cells, and high concentrations of AR ligands exhibit a bipolar effect where prostate carcinoma cell growth is attenuated at both high and low levels of androgens (12, 74). We confirmed that high AR levels repress LNCaP_FGC cell proliferation using a doxycycline-inducible AR construct. After 12 days of growth, the induction of AR reduced LNCaP_FGC cell numbers from 50% to 20% of the population (adjusted *P* = 8 × 10^–6^) (Supplemental Figure 6, F–I). Given that AR-overexpressing LNCaP_AR909 cells proliferate well in standard medium, these data suggest that a component of the gene expression alterations evident in LNCaP_AR909 cells are associated with the ability to tolerate high AR levels and maintain a proliferative drive, potentially by shifting cell lineage commitment from a terminally differentiated luminal cell phenotype. In support of this conclusion, LNCaP_AR909 cells expressed higher levels of KLF5 and a KLF5 transcriptional program, which has been shown to oppose AR activity and promote cell migration and a basal epithelial cell–like phenotype. In addition to diminished expression of the luminal cytokeratin KRT8, the LNCaP_AR909 cells expressed features of adult SLCs and lineage pathways including WNT, Notch, and epithelial-mesenchymal transition (Figure 8, C–E). Of interest, although the AR cistrome and components of the AR program were attenuated in LNCaP_AR909 cells, they retained the phenotype of growth repression by supraphysiological androgens (Supplemental Figure 6J).

Prior studies using the LNCaP_AR909 model to identify drivers of enzalutamide insensitivity determined that loss of *chromodomain helicase DNA binding protein 1* (CHD1) promoted diverse pathways of resistance via the upregulation of 4 transcription factors: NR3C1, POU3F2, NR2F1, and TBX2, which also associated with the loss of luminal epithelial differentiation (47). We found that even without *CHD1* loss or exposure to AR signaling inhibitors, the LNCaP_AR909 cells had differential upregulation of POU3F2, NR2F1, and TBX2 (Figure 8E). As described below, LNCaP_ABL cells express high levels of NR3C1/GR but have no alterations in *CHD1*. Collectively, these results suggest that the inherent heterogeneity and plasticity of LNCaP cells provide diverse nongenomic mechanisms for overcoming proliferation constraints related to AR pathway repression or hyperactivation.

### LNCaP strains exhibit differential drivers and dependencies relevant for metastatic prostate carcinoma.

**Figure 9:**
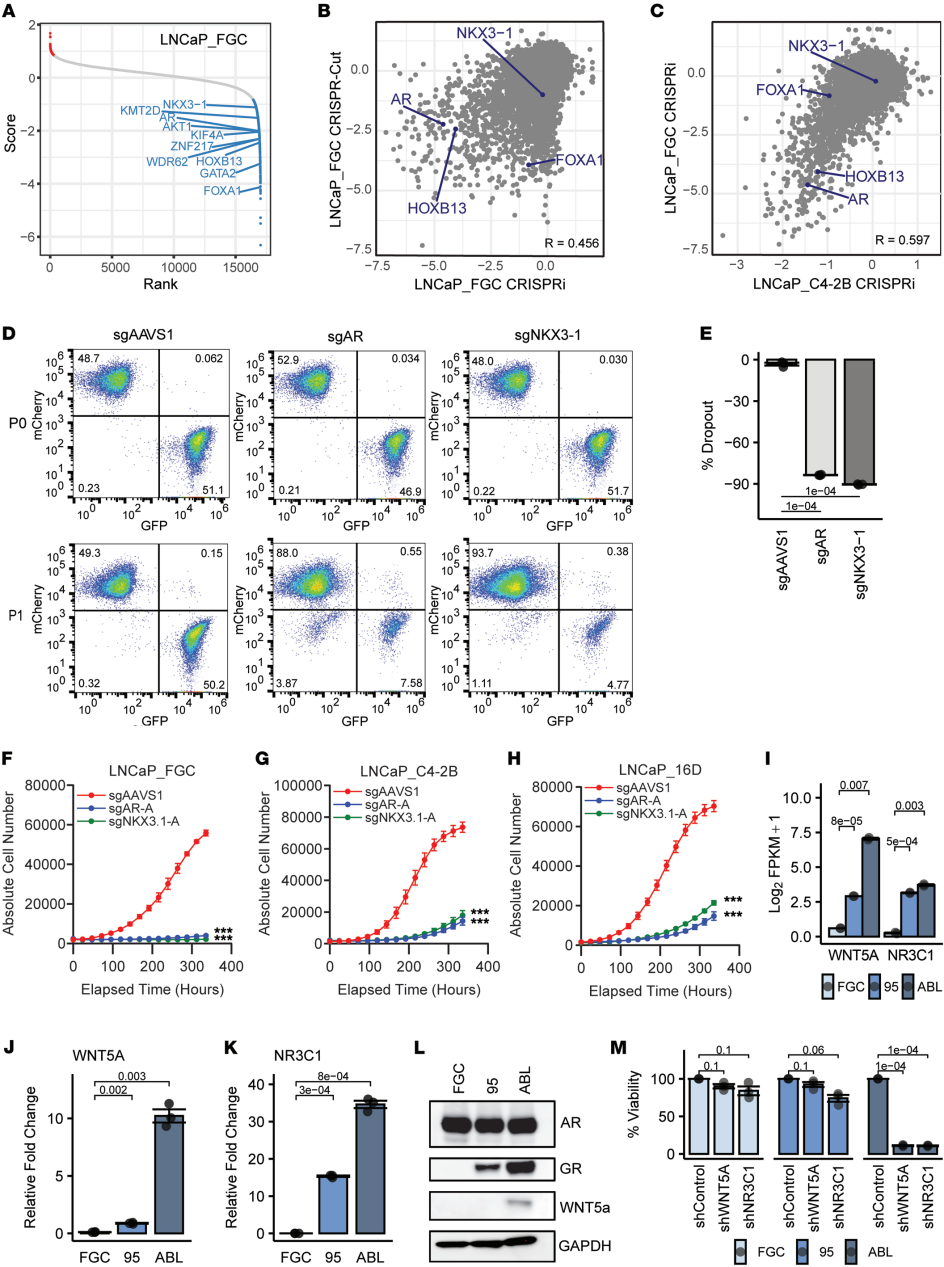
LNCaP substrains exhibit differential drivers and dependencies relevant for metastatic prostate carcinoma. (**A**) CRISPR/Cas9 whole-genome knockout/depletion screen in LNCaP_FGC cells. (**B**) Comparison of gene dependencies in LNCaP_FGC identified by CRISPR/Cas9 deletion (CRISPR-Del) screen versus a previously reported CRISPR interference (CRISPRi) screen. (**C**) Comparison of gene dependencies in LNCaP_FGC versus LNCaP_C4-2B determined by CRISPRi screens. (**D**) Competition assay of LNCaP_FGC cells assessing the effects of a safe harbor control locus (AAVS1), *AR* gene deletion, and *NKX3.1* gene deletion on cell viability. P0 represents the initial time point of mixing of mCherry (control) and GFP (sgAR or sgNKX3-1) cells, and P1 is cell numbers measured after 12 days of growth. (**E**) Quantification of the percentage change of GFP^+^ population for the indicated sgRNAs in **D** for P1 time point with respect to P0. Groups compared by unpaired *t* tests with Benjamini-Hochberg–adjusted *P* values shown on plot. (**F**–**H**) Growth curves of LNCaP_FGC, LNCaP_C4-2B, and LNCaP_16D following CRISPR/Cas9 deletion of NKX3.1 and AR versus control. Growth curves comparing cell numbers of control (sgAAVS1) versus experimental (sgAR and sgNKX3.1) cells after 350 hours were fit and compared by nonlinear regression (***P < 0.0001). (**I**) Transcript abundance by RNA-Seq of WNT5A and NR3C1/GR in LNCaP_FGC, LNCaP_95, and LNCaP_ABL (*n* = 2 per line). Groups compared by unpaired *t* tests with Benjamini-Hochberg–adjusted *P* values shown on plot. (**J**) WNT5A and (**K**) NR3C1/GR transcript abundance by qRT-PCR in LNCaP_FGC, LNCaP_95, and LNCaP_ABL cells grown in steady-state conditions (*n* = 3 per line). Groups compared by unpaired *t* tests with Benjamini-Hochberg–adjusted *P* values shown on plots. (**L**) Immunoblot of WNT5A and NR3C1/GR protein in LNCaP_FGC and substrains. (**M**) Influence of NR3C1 and WNT5A repression by shRNA on the viability of LNCaP_FGC, LNCaP_ABL, and LNCaP_95 (*n* = 3 per line). Groups compared by unpaired *t* tests with Benjamini-Hochberg–adjusted *P* values shown on plot.

The diversity of genomes and transcriptomes across LNCaP substrains suggested that functional studies of differential alterations in these models could provide insights into mechanisms responsible for clinical outcomes and therapeutic responses observed in patients with metastatic prostate carcinoma. To gain an initial assessment of molecular dependencies, we performed a whole-genome CRISPR/Cas9 deletion screen in the LNCaP_FGC line. Overall, using a cutoff of 2-fold depletion, 722 genes met the criteria for growth or survival dependency. Of these, 607 annotated as “common essential” across multiple cancer cell line models screened in the Dependency Map (DepMap) portal (https://depmap.org/portal/). In addition to common essential genes, genes with known relevance to prostate carcinoma pathobiology, including *AR*, *FOXA1*, *HOXB13*, *GATA2*, *SPOP*, and *AKT,* were depleted (Figure 9A). We compared the CRISPR results here with a prior study reported by Das et al. using a CRISPRi (dCAS9-KRAB) whole-genome screen to assess LNCaP dependencies (Figure 9B) (75). Two highlighted hits, KIF4A and WDR62, reported to influence prostate carcinoma survival and aggressive behavior, were also identified in our LNCaP_FGC screen (Figure 9A). Das et al. (75) also conducted a CRISPRi screen of LNCaP_C4-2B, and a comparison of hits between LNCaP_FGC and LNCaP_C4-2B showed generally high concordant dependencies (*r* = 0.60) (Figure 9C).

Of interest, several sgRNAs targeting the *NKX3.1* gene were depleted in the LNCaP_FGC CRISPR screen, suggesting that loss of NKX3.1 is detrimental to prostate carcinoma survival or growth (Figure 9A). This result was unexpected because *NKX3.1* is widely considered to exhibit prostate carcinoma tumor suppressor activity. *NKX3.1* encodes a prostate-specific homeobox gene with functions that mediate AR signaling and influence normal prostate developmental processes (76). Genomic loss of the NKX3.1 locus is a common event in prostate carcinoma, and genetically engineered mouse models exhibit elevated rates of neoplasia when *Nkx3.1* is deleted (77, 78). However, genomic loss of *NKX3.1* is usually monoallelic and complete loss in prostate carcinoma is extremely rare, suggesting important contributions to prostate carcinoma viability (79). Notably, NKX3.1 did not score in the DepMap LNCaP_FGC data (80), and though depleted in the CRISPRi screens of LNCaP_FGC and LNCaP_C4-2B, it did not reach significance. Consequently, we investigated the performance of individual sgRNAs targeting NKX3.1 and found a wide variance, with only 2 of 4 guides in the whole-genome CRISPR screen showing 3-fold depletion (Supplemental Figure 6K). LNCaP_APIPC cells that are AR-null and do not express NKX3.1 were not affected by sgRNAs targeting NKX3.1 (Supplemental Figure 6K). To further investigate the potential dependency of prostate carcinoma on NKX3.1, we performed a competition assay using independent sgRNAs targeting NKX3.1 and confirmed that NKX3.1 loss significantly reduced the viability of LNCaP_FGC and other LNCaP strains to a level that approximated the effects of AR deletion (Figure 9, D–H).

The comparative assessments of LNCaP transcriptomes identified substrain-specific features with the potential to regulate prostate carcinoma phenotypes such as resistance to ADT and ARSI therapy (Figure 3). Notably, by RNA-Seq quantitation, transcripts encoding WNT5A were increased 5-fold (adjusted *P* = 8 × 10^–5^) and 87-fold (adjusted *P* = 0.007) in castration-resistant LNCaP_95 and LNCaP_ABL cells, respectively, compared with LNCaP_FGC. Similarly, the expression level of the glucocorticoid receptor GR/NR3C1 was increased 7.4-fold (adjusted *P* = 5 × 10^–4^) in LNCaP_95 and 11-fold (adjusted *P* = 0.0003) in LNCaP_ABL relative to LNCaP_FGC (Figure 9I). We confirmed the differential expression of WNT5A and NR3C1 by qRT-PCR, IHC, and immunoblot (Figure 9, J–L, and Supplemental Figure 7). Repression of WNT5A and GR/NRC3C1 had no effect on the growth of LNCaP_FGC. In contrast, knockdown of NR3C1 reduced the viability of LNCaP_95 by 26% (adjusted *P* = 0.06) and LNCaP_ABL by 89% (adjusted *P* = 0.0001), and suppression of WNT5A reduced the viability of LNCaP_ABL by 89% (adjusted *P* = 0.0001) (Figure 9M and Supplemental Figure 6L).

## Discussion

The LNCaP cell line has filled a major void in the cancer research field where clinically relevant models of prostate carcinoma genotype and phenotype are scarce (81, 82). The remarkable versatility of the LNCaP line has enabled a broad spectrum of research applicable to human prostate carcinoma, including studies of oncogenic drivers, drug resistance, metastatic potential, and lineage plasticity. However, as studies of other cancer cell lines — such as MCF7 breast cancer — have revealed (83), it is critical to recognize the heterogeneity, clonal dynamics, and continuous instability inherent in these models, which reflects ongoing processes that also occur in human hosts. These observations are likely also relevant to cancer xenografts and organoid systems, emphasizing the need to replicate findings across multiple models to ensure rigor and the accuracy of conclusions.

Comparative analyses of cell strains derived from parental LNCaP_FGC provide a number of interesting observations with respect to oncogenic processes in general and prostate carcinoma pathobiology specifically. We identified the presence of a subclonal deleterious *TP53* mutation representing only 0.15% of the genomic reads sampled in the parental LNCaP_FGC line used in the present study. The frequency of this mutation is elevated in various LNCaP-derived strains, but in no strain does it progress to clonal dominance despite years of propagation or therapeutic pressure. Loss of the Y chromosome has been shown to result in oncogenic effects, yet Y chromosome loss remained subclonal in several LNCaP substrains. Of specific relevance to prostate carcinoma, the AR plays a central role for the survival and growth of prostate carcinoma and consequently has served as a key target for therapeutic intervention. Clinical studies have identified multiple mechanisms — sometimes occurring in the same patient — that contribute to the maintenance of AR signaling, including AR LBD mutations, AR splice variants, and amplification of the *AR* and *AR* enhancer locus (25, 57, 84). However, it is interesting that LNCaP substrains do not universally activate new resistance mechanisms operating through the AR, despite intense therapeutic pressures. Collectively, evidence for convergent evolution to activate the AR program is evident, but only LNCaP_95 expresses ARv7 to any appreciable extent, and only one strain, LNCaP_42D, has a new structural alteration involving AR locus amplification. Although prior studies demonstrate that additional AR mutations can arise in LNCaP with extremely low frequencies (85), these observations suggest that the T878A AR LBD mutation is able, under most in vitro and in vivo growth conditions, to maintain a level of AR activity sufficient to promote the survival of at least a subpopulation of LNCaP_FGC cells that allow the eventual emergence of clones or substrains capable of growth after ADT or ARSI therapy. Notably, even under androgen-depleted conditions or AR antagonists, all of the LNCaP substrains retain AR activity and do not fully transdifferentiate to AR-null phenotypes as a resistance mechanism.

Gene rearrangements involving members of the ETS oncogene family are observed in 30%–50% of prostate carcinomas and have been shown to promote the development of prostate carcinoma in model systems (86–88). A gene rearrangement involving *ETV1* is found in LNCaP_FGC, and we confirmed this event is present in all of the LNCaP substrains. However, ETV1 is only expressed in the parental LNCaP_FGC line and one strain, LNCaP_ABL. Further, in LNCaP_FGC, single-cell analysis indicated that ETV1 expression was quite heterogeneous. A comparative analysis of the Chr14-7 locus of rearrangement did not identify genomic differences that would explain differential expression, nor did we observe a difference in chromatin accessibility. Notably, the LNCaP_C4-2B strain, which was derived from bone metastasis resulting from a subcutaneous LNCaP_C4 implant (10), lacks ETV1 expression, indicating that ETV1 may be dispensable for metastatic behavior, at least in murine hosts.

Although all of the LNCaP strains share key attributes with LNCaP_FGC, the distinctive differences in the gene expression, chromatin accessibility, and AR cistromes between the LNCaP strains indicates that features of their propagation conditions, including in vivo growth and drug exposures, have shaped their phenotypes. Overall, the development of these diverse strains that represent relevant features of human prostate carcinoma attests to the remarkable versatility of LNCaP_FGC as a foundational platform. The inherent genomic heterogeneity and phenotypic plasticity of LNCaP allows for the emergence of diverse epigenetic resistance mechanisms, as exemplified by prior studies of enzalutamide-tolerant LNCaP subclones with apparent divergence in transcription factor drivers spanning *NR3C1*, *POU3F2*, *NR2F1*, and *TBX2* that are confirmed in studies of human metastatic CRPC (14, 46, 47).

Although clonal diversity, genomic instability, and epigenetic plasticity have virtues in promoting versatility, these features should be recognized to also have consequences for the accurate interpretation of an experimental result and the reproduction of findings across research groups. Issues underscoring a crisis in reproducibility have been well documented (89), and the data presented here clearly demonstrate that one contributing factor centers on cell line and cell strain heterogeneity that may be completely unrecognized in the design of experiments. These data indicate that “Your LNCaP is not my LNCaP” because contemporary isolates of LNCaP_FGC obtained directly from reputable repositories will inherently comprise heterogenous populations of cells. We propose that it would be useful for repositories to develop single-cell clonal lines of LNCaP_FGC and substrains to serve as a more consistent baseline for the research community, recognizing that the ongoing mutagenesis processes will result in divergence over time. Further, the similarity between the genomes of LNCaP-derived strains challenge the use of STR genotyping for authentication. To this end, the panel of strain-specific SNVs we identified can serve this purpose when interrogating a large component of the genome via RNA-Seq, WES, or WGS.

“Out of one, many” is a reasonable description for the LNCaP cell line. Although few models of prostate carcinoma exist, the diverse LNCaP substrains provide substrate for mechanistic studies to address key biological questions relating to clinically relevant biology, including DNA mismatch repair deficiency, the genesis of AR splice variants, processes driving AR amplification, consequences of TP53 inactivation, the role of Y chromosome loss, the contribution of ETS-transcription factors to prostate carcinoma pathobiology, and systemic screens to identify prostate carcinoma vulnerabilities in the context of lineage plasticity.

## Methods

### Sex as a biological variable.

Prostate cancer only occurs in males, and consequently all models evaluated were derived from men.

### Experimental methods.

Full details on the methods used in the studies are provided in the Supplemental Methods.

### Statistics.

Statistical analyses pertaining to each figure are included within the figure legends. For comparisons of distributions of categorical variables, we performed Fisher’s exact test using Benjamini-Hochberg multiple-testing correction in R. Continuous variables were compared between groups with unpaired *t* tests using Benjamini-Hochberg multiple-testing correction in R. Pearson’s correlation coefficient computed in R was used to study the relationships between variables shown in scatterplots. Growth curves were fit and compared by nonlinear regression in GraphPad Prism10.3.1. A *P* value <0.05 was considered significant.

### Study approvals.

All experiments were carried out in accordance with the Fred Hutchinson Cancer Center’s approved protocol IR 6312. No human subjects or vertebrate animals were used in the studies.

### Data availability.

The Supporting data values for each figure and panel are included in the table titled Supporting Data Values. The RNA-Seq data, ATAC-Seq data, and AR ChIP-Seq data have been deposited in NCBI’s Gene Expression Omnibus (GEO) under the accession numbers GSE288591, GSE288843, GSE288878, GSE289031, and GSE289398. The WGS data are deposited under accession number PRJNA1219540 in the sequence read archive. The raw data used in this study will be shared by Peter S. Nelson upon request. Additional information and methods/code required to analyze the data in this study are available on GitHub and/or will be provided upon request. Additional information and requests for resources and reagents should be sent to Peter S. Nelson.

## Author contributions

A Bose, WH, TC, JL, DL, RB, and RD designed research studies and conducted experiments. A Bankhead, IC, TP, BH, PI, SH, MB, MK, and CP conducted experiments, acquired data, and analyzed data. PG and MH acquired and analyzed data. GH and PSN designed research studies, analyzed data, and wrote the initial drafts of the manuscript. All authors reviewed and edited the final manuscript.

## Funding support

This work is the result of NIH funding, in whole or in part, and is subject to the NIH Public Access Policy. Through acceptance of this federal funding, the NIH has been given a right to make the work publicly available in PubMed Central.

- P30CA015704, P50CA097186, P01CA163227, DP2CA280624, R01CA280056, R01CA266452, R01CA234715, R21 CA277368, and R50CA274336 from the NIH.
- PC230420, PC230582, PC230599, PC220456, PC240661, and PC220456 from the Department of Defense.
- The Prostate Cancer Foundation and the Institute for Prostate Cancer Research.

## Supplementary Material

Supplemental data

Unedited blot and gel images

Supplemental table 1

Supplemental table 2

Supplemental table 3

Supplemental table 4

Supporting data values

## Version 1 09/16/2025

In-Press Preview

## Version 2 11/17/2025

Electronic publication
