# Supplementary material for: Multiomic assessments of LNCaP and derived cell strains reveal determinants of prostate cancer pathobiology
Source: J Clin Invest. 2025 Sep 16;135(22):e194727. doi: 10.1172/JCI194727 (PMC12618073; doi:10.1172/JCI194727)
Supplement: Supplemental data [file jci-135-194727-s096.pdf]

Multiomic assessments of LNCaP and derived cell strains reveal determinants of prostate cancer pathobiology

Bose et al.

SUPPLEMENTARY MATERIALS

Supplementary Figures S1-7

Supplementary Tables
Supplementary Table S1. Metrics of the LNCaP_FGC genome determined by WGS.
Supplementary Table S2. Metrics of LNCaP substrain genomes determined by WGS
Supplementary Table S3. Mutations and structural alterations in LNCaP substrain genomes.
Supplementary Table S4. Gene expression features of LNCaP substrain transcriptomes.

Supplementary Methods

Supporting Data Values Table

Original Gel Images for Immunoblots

Supplementary Figure 1

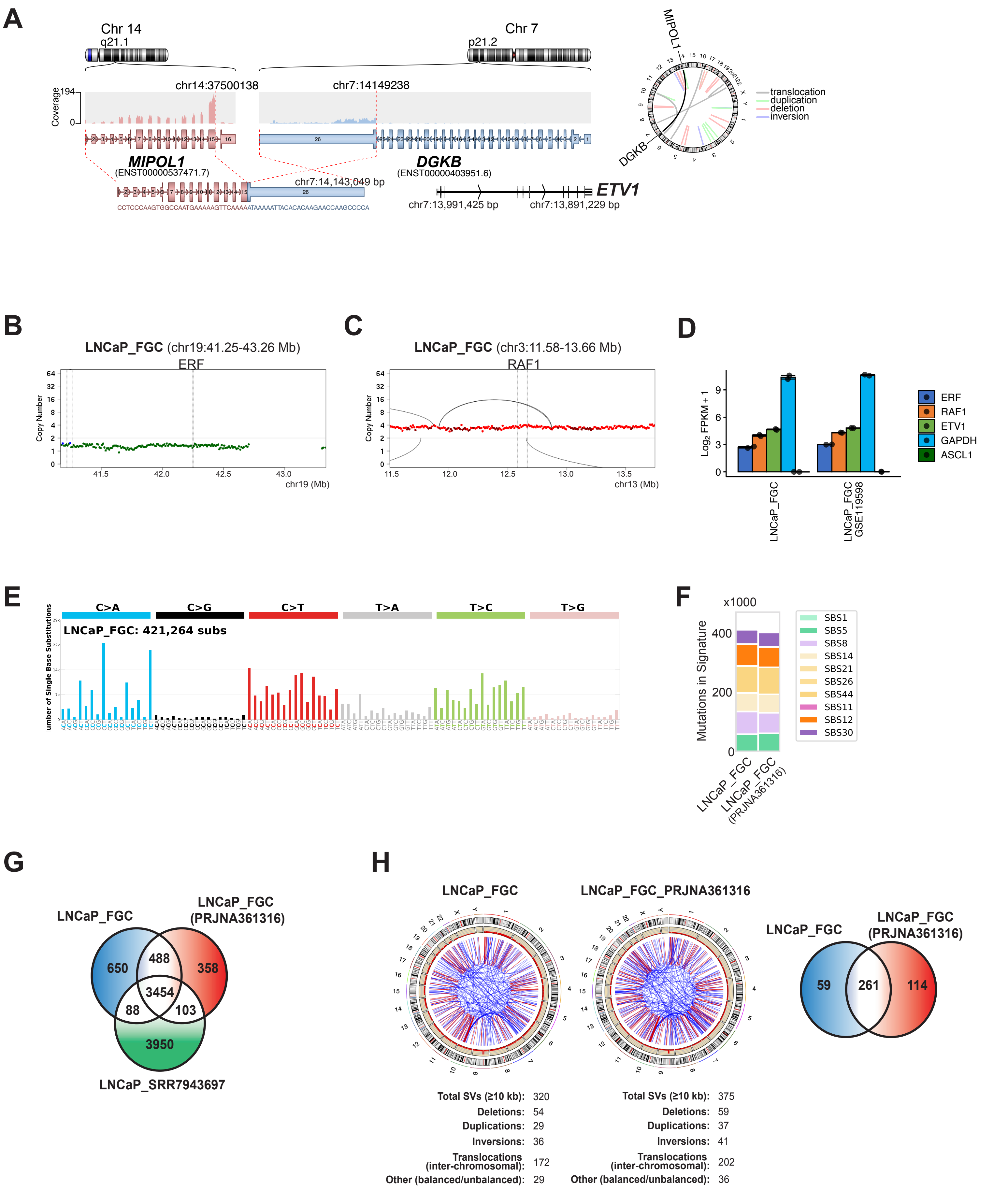

Supplementary Figure S1. Genomic alterations and oncogenic events in the LNCaP_FGC genome

(A) Genomic rearrangement and downstream fusion between *ETV1* and *MIPOL1* observed within the LNCaP parental line (FGC) where *MIPOL1* has been inserted into *DGKB*. Arriba used both genome (WGS) and transcriptome data for fusion analysis and to generate this visualization. The *ETV1* gene model was included manually for reference.

(B) Copy number profile in the region of chr 19 (41.25-43.26 Mb) containing *ERF* (gene boundaries indicated with vertical dotted lines). Data points represent germline heterozygous SNPs or genomic bins (10 kbps). Green indicates deletion.

(C) Copy number profile in the region of chr 3 (11.58-13.66 Mb) containing *RAF1* (gene boundaries indicated with vertical dotted lines). Data points represent germline heterozygous SNPs or genomic bins (10 kbps). Shades of red indicates copy gain or amplification. Arc lines represent genomic rearrangements (intrachromosomal events on top; interchromosomal events on bottom).

(D) Transcript abundance by RNAseq of selected genes in LNCaP_FGC (this study) and LNCaP_FGC from GSE119598 (n=2 per line.)

(E) Single base substitution counts in a tri-nucleotide (left and right flanking base) context for LNCaP_FGC. All 409,210 base substitutions (SNVs) are included.

(F) Distribution of SNVs assigned to each single base substitution signature (SBS). COSMIC signatures as analyzed by SigProfiler analysis is shown.

(G) Comparison of all coding SNVs between three LNCaP whole genome sequence studies: LNCaP_FGC (this study), and two published WGS analyses: LNCaP_FGC_PRJNA361316, and LNCaP_SRR7943697.

(H) Circos plots illustrating structural variants (SV) events for LNCaP_FGC and LNCaP_FGC_PRJNA361316. Red lines, intra-chromosomal SVs; blue lines, inter-chromosomal SVs. Comparison of SVs between the lines shown in the Venn diagram (right) was based on overlapping SV events. An overlap of two SVs, one from each sample used criteria: (1) leftmost breakpoints overlap within 200 bp for both SVs, and (2) rightmost breakpoints overlap within 200 bp for both SVs, and (3) 80% reciprocal overlap of both SVs by their genomic span.

Supplementary Figure 2

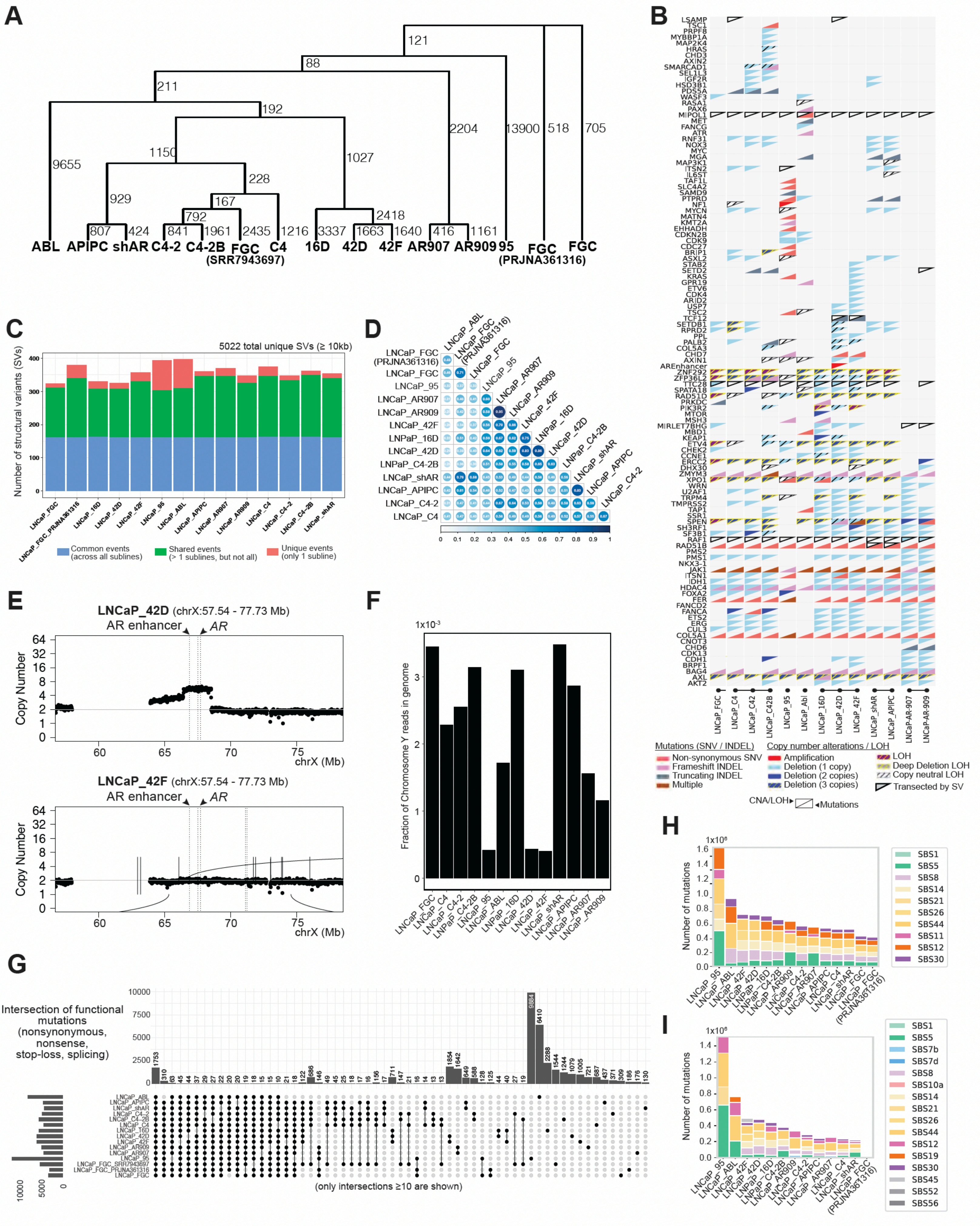

Supplementary Figure S2. LNCaP sublines exhibit recurrent and unique genomic alterations.

(A) Phylogenetic tree for LNCaP substrains based on all unique coding (including splicing) SNV mutations, (48,288) across the substrains. The phylogram was generated using neighbor-joining tree estimation; Manhattan/Hamming distance metric was used for binary status of the mutations. The edges in the tree indicate the number of mutations; however, the length of the edges is not draw to scale.

(B) Mutation and copy number alteration (CNA) status for selected genes relevant to prostate cancer (not already shown in Fig. 1E) across LNCaP strains. The top left triangle indicates an observed pathogenic mutation (SNV/INDEL) within the gene and the bottom right triangle indicates CNA status (Amplification, Shallow Deletions, Deletions, Deep Deletions, and Homozygous Deletions) that overlap the gene. CNA events that also have loss of heterozygosity (LOH) status are depicted with a hatch pattern. 'Multiple' indicates instances where 2+ mutations are observed for that gene. Tumor mutation burden was computed as the number of non-synonymous mutations per megabase pairs of coding regions (top). Aneuploidy status (Arm-gain, Arm-deletion) is indicated for select chromosome arms. Fusions status indicates evidence for genomic rearrangement involving an ETS transcription factor. Genes that have been transected by at least one of two breakpoints of a structural variation (SV) event is indicated with a black border at the top left triangle.

(C) Number of structural variants (SVs) that are ≥ 10 kb in the LNCaP sublines. SV event counts are shown based on detection in all sublines (blue), shared in >1 subline but not in all (green), and unique to just a single subline (red).

(D) Gene-level copy number analysis between LNCaP sublines. Pearson's correlation coefficient is shown between all pairs of sublines based on comparing integer copy number. 19,467 protein coding genes were included based on GRCh38.p12 Ensembl gene annotations and having aberrant copy number beyond 4 ploidy in at least one subline.

(E) Copy number comparison in the AR region of chromosome X (57.54 -77.73 Mb; GRCh38) for LNCaP_42D and LNCaP_42F. AR gene and the AR enhancer are indicated with vertical dotted lines. Data points represent germline heterozygous SNPs or genomic bins (10 kbps). Arc lines represent genomic rearrangements (intrachromosomal events on top; interchromosomal events on bottom).

(F) Fraction of chromosome Y reads across LNCaP sublines. The fraction of chromosome Y reads was computed as the number of chromosome Y reads divided by the total number of reads in all autosomes for that given subline.

(G) Upset plot for the presence and intersection of functional mutations (SNVs), which includes non-synonymous, nonsense, stop-loss, and splicing mutations. Comparisons between substrain combinations are only shown when the number of intersecting mutations is ≥10.

(H) Mutation signatures identified for LNCaP FGC and all sublines. All mutations were used after filtering out germline events from with >10% frequency in gnomAD or ExAC databases. Mutations were called independently (tumor-only mode) for each sample.

(I) Mutation signatures identified for LNCaP FGC and all substrains, using mutations called for each sub-line relative to LNCaP_FGC (FGC was used as the "normal" sample in the analysis).

Supplementary Figure S3

A

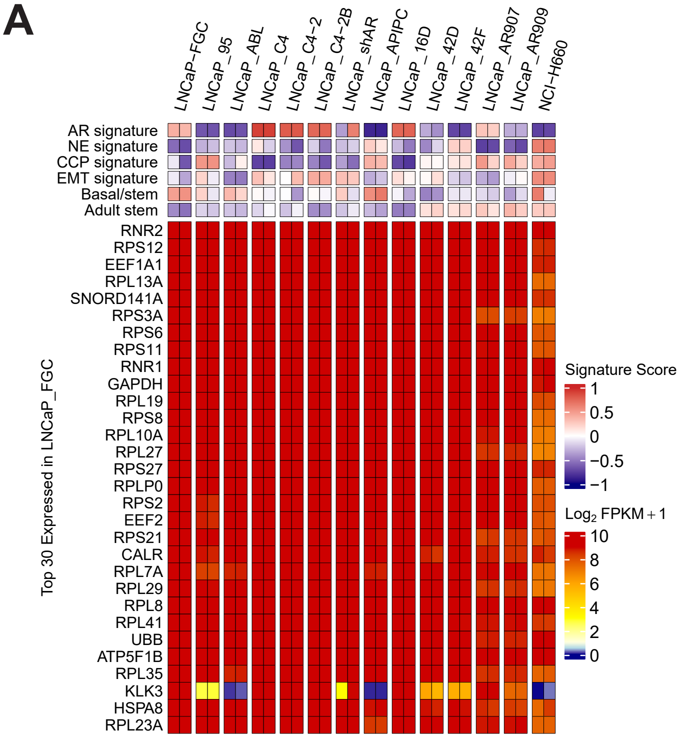

B

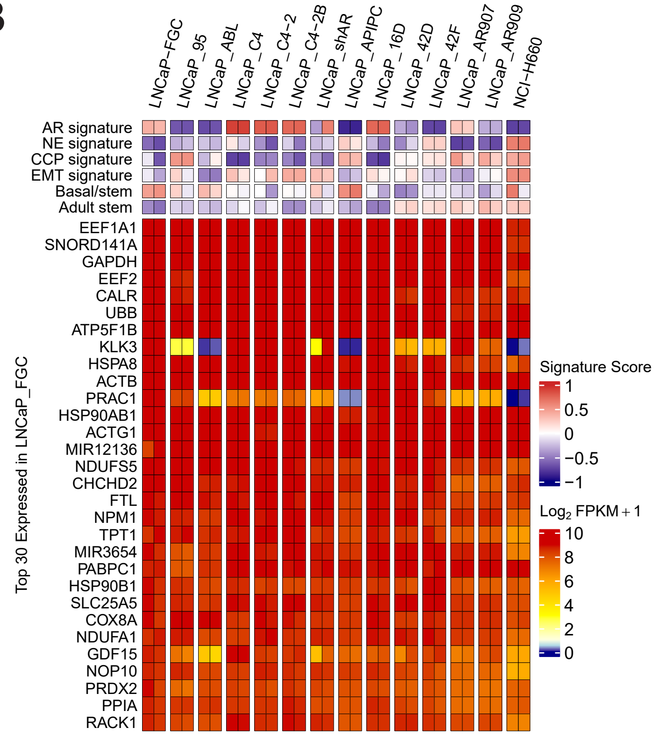

C

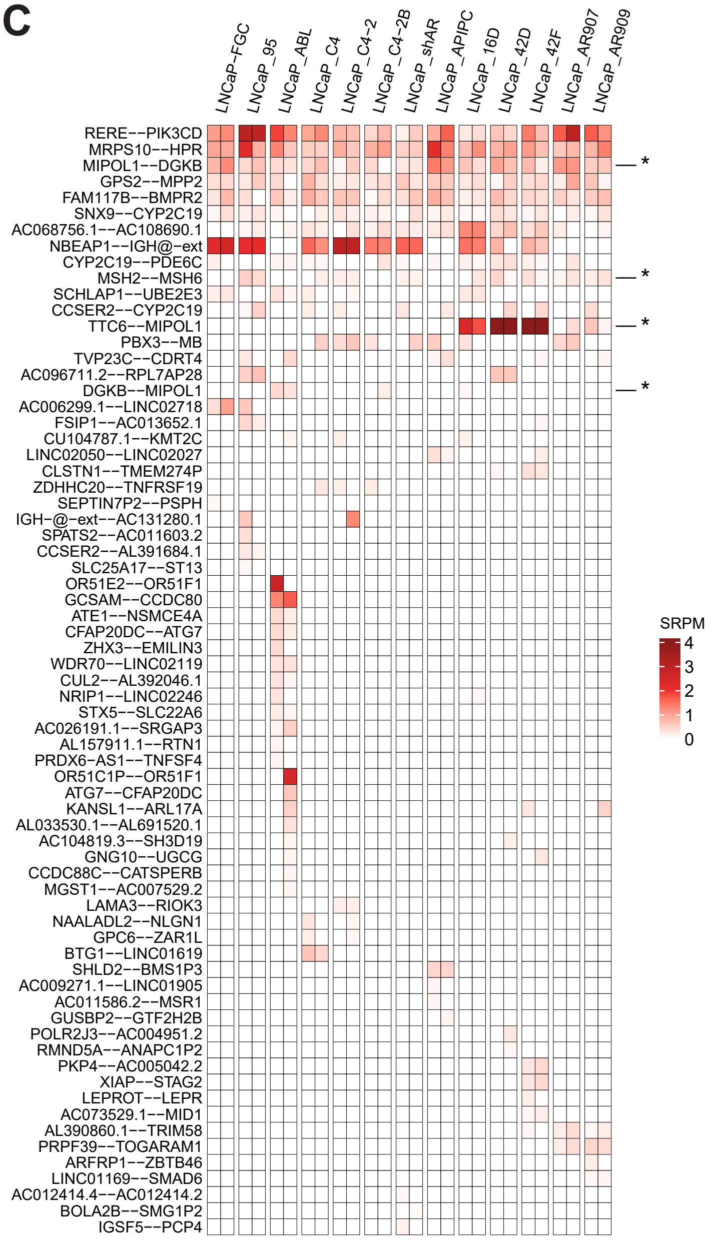

D

Line	FDR<0.05, FC>3	FDR<0.05, FC<-3
LNCaP_FGC	439	790
LNCaP_95	988	1020
LNCaP_ABL	1086	1403
LNCaP_C4	513	535
LNCaP_C4-2	500	446
LNCaP_C4-2B	536	639
LNCaP_shAR	608	295
LNCaP_APIPC	1265	643
LNCaP_16D	559	683
LNCaP_42D	926	670
LNCaP_42F	1266	815
LNCaP_AR907	582	1216
LNCaP_AR909	594	1004

E

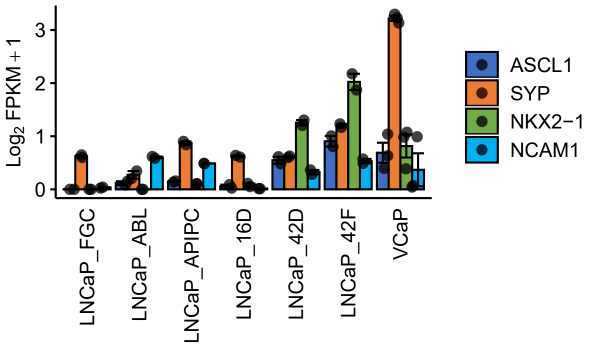

F

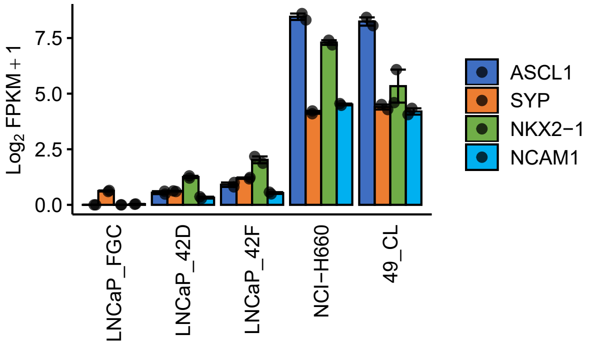

Supplementary Figure S3. LNCaP substrains express divergent transcriptomes.

(A) Heatmap of transcript abundances of the top 30 genes expressed in LNCaP_FGC. GSVA signature scores and log2 FPKM values are colored according to scales shown on plot. AR: androgen receptor, NE neuroendocrine, CCP: cell cycle progression, EMT: epithelial-mesenchymal transition,

(B) Heatmap of transcript abundances of the top 30 genes expressed in LNCaP_FGC after the exclusion of ribosomal genes.

(C) Heatmap of gene fusion expression across LNCaP strains, by spliced reads per million (SRPM) color scale. Selected gene fusions are indicated with asterisks.

(D) Table of number of differentially expressed genes in pairwise comparisons between each line and remaining 12 other strains.

(E) Transcript abundance by RNAseq of selected NE phenotype genes across different cell strains (n=2 per line.)

(F) Transcript abundance by RNAseq of selected NE phenotype genes across different cell strains and NCI-H660 and LUCaP49_CL neuroendocrine prostate cancer (NEPC) cell lines (n=2 per line.)

Supplementary Figure S4

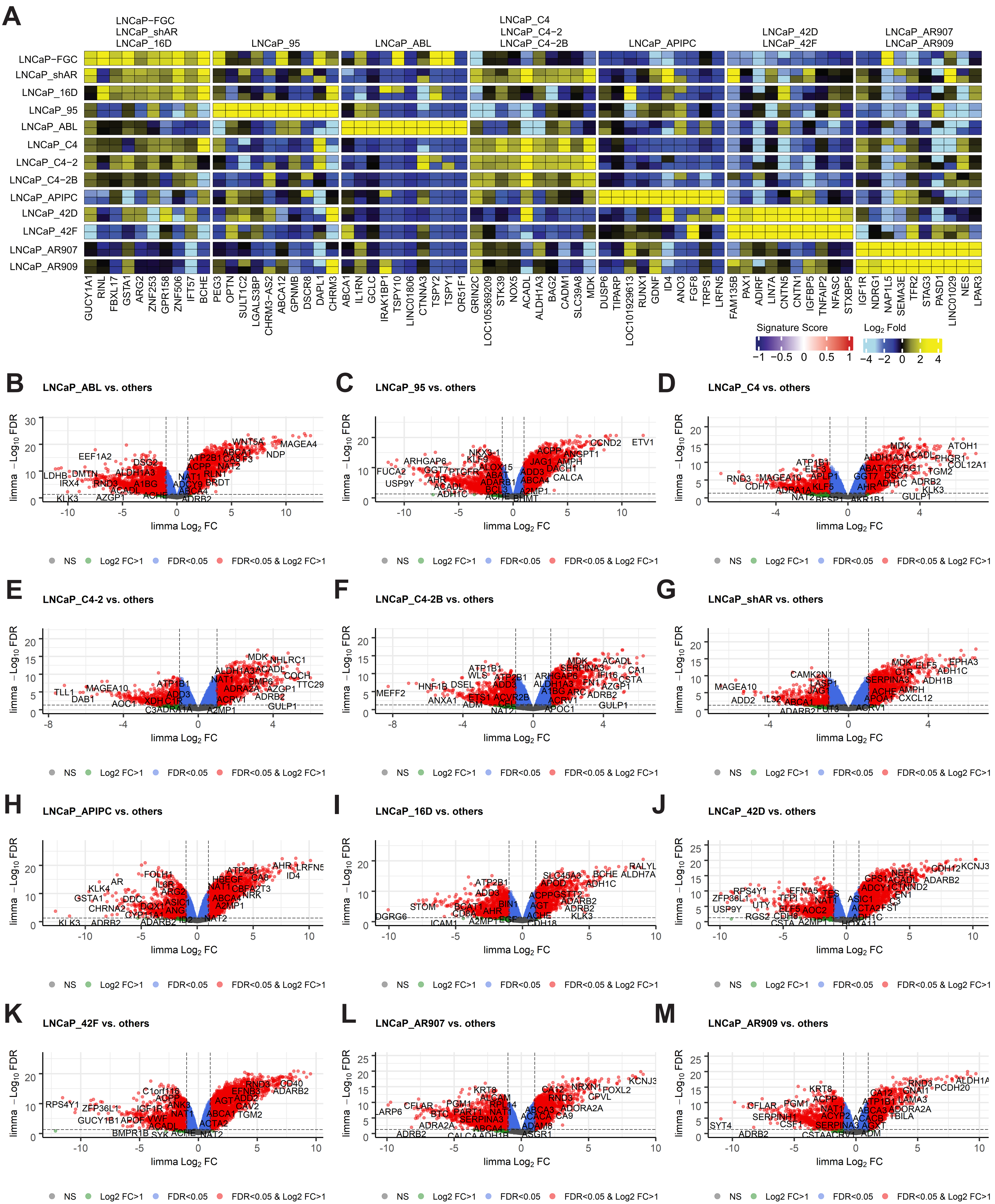

Supplementary Figure S4. LNCaP substrains express divergent transcriptomes.

(A) Heatmap of the top 10 genes differentially increased in related LNCaP substrains compared to other related substrains with FDR<0.05. GSVA (gene set variation analysis) scores and log2 relative fold ratios are colored according to scales shown on plot.

(B-M) Volcano plots of differential expression analysis between each designated line/strain compared to all other substrains.

Supplementary Figure S5

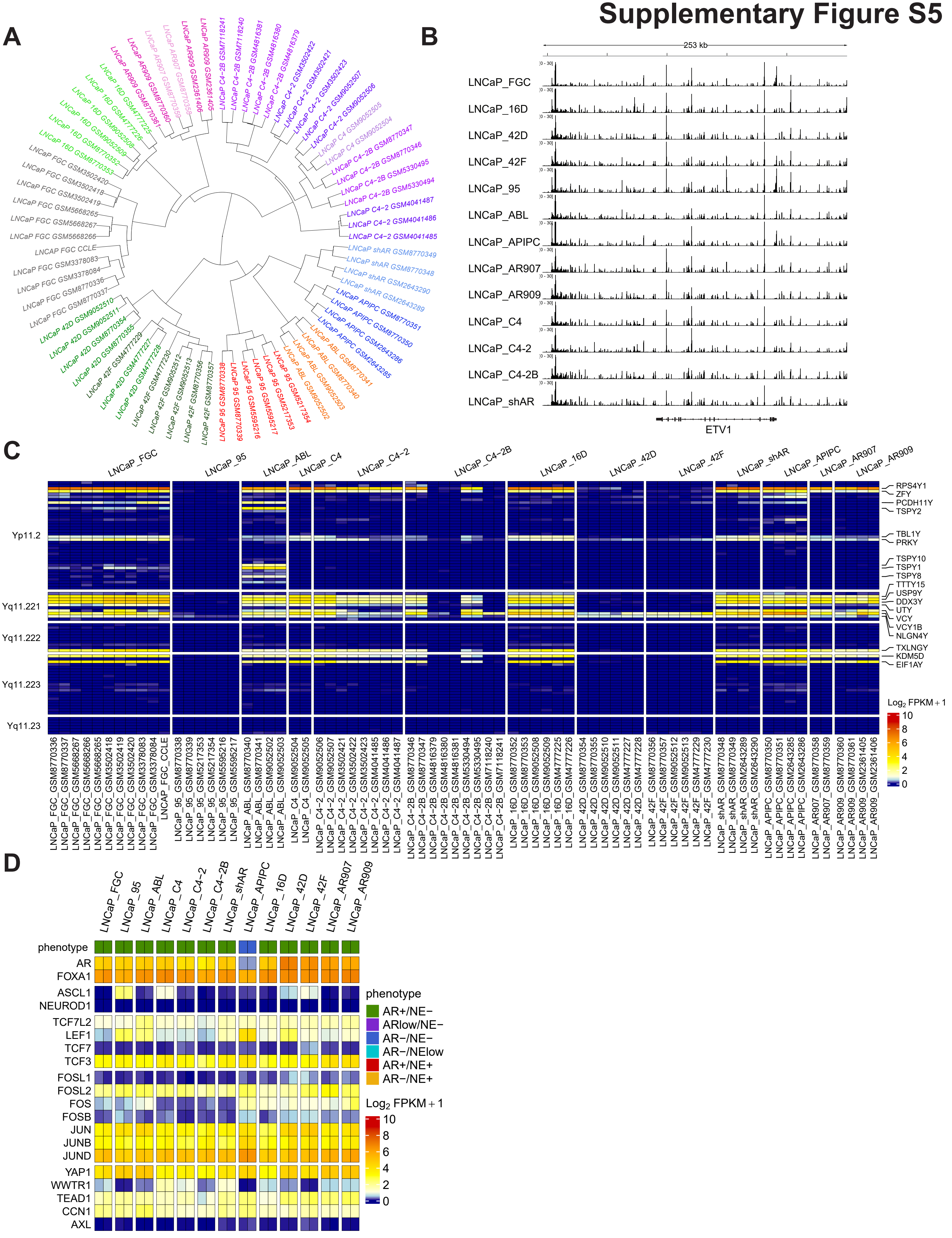

Supplementary Figure S5. LNCaP substrains express divergent transcriptomes.

(A) Hierarchical clustering of transcript data generated in this study and public datasets of LNCaP_FGC and substrains, using the top 1000 most variable genes, Ward's D2 method, and Euclidian distance. Samples are colored according to line/strain.

(B) ATACseq peaks mapping to the *ETV1* locus across LNCaP strains.

(C) Heatmap of transcript abundances of genes on the Y-chromosome across data generated in this study and public datasets of LNCaP_FGC and substrains. Log2 FPKM values are colored according to scales shown on plot. Genes with detectable expression in at least one sample are listed on the right side of the plot.

(D) Heatmap of transcript levels encoding transcription factors associated with prostate cancer AR, NE, SCL, WNT lineages.

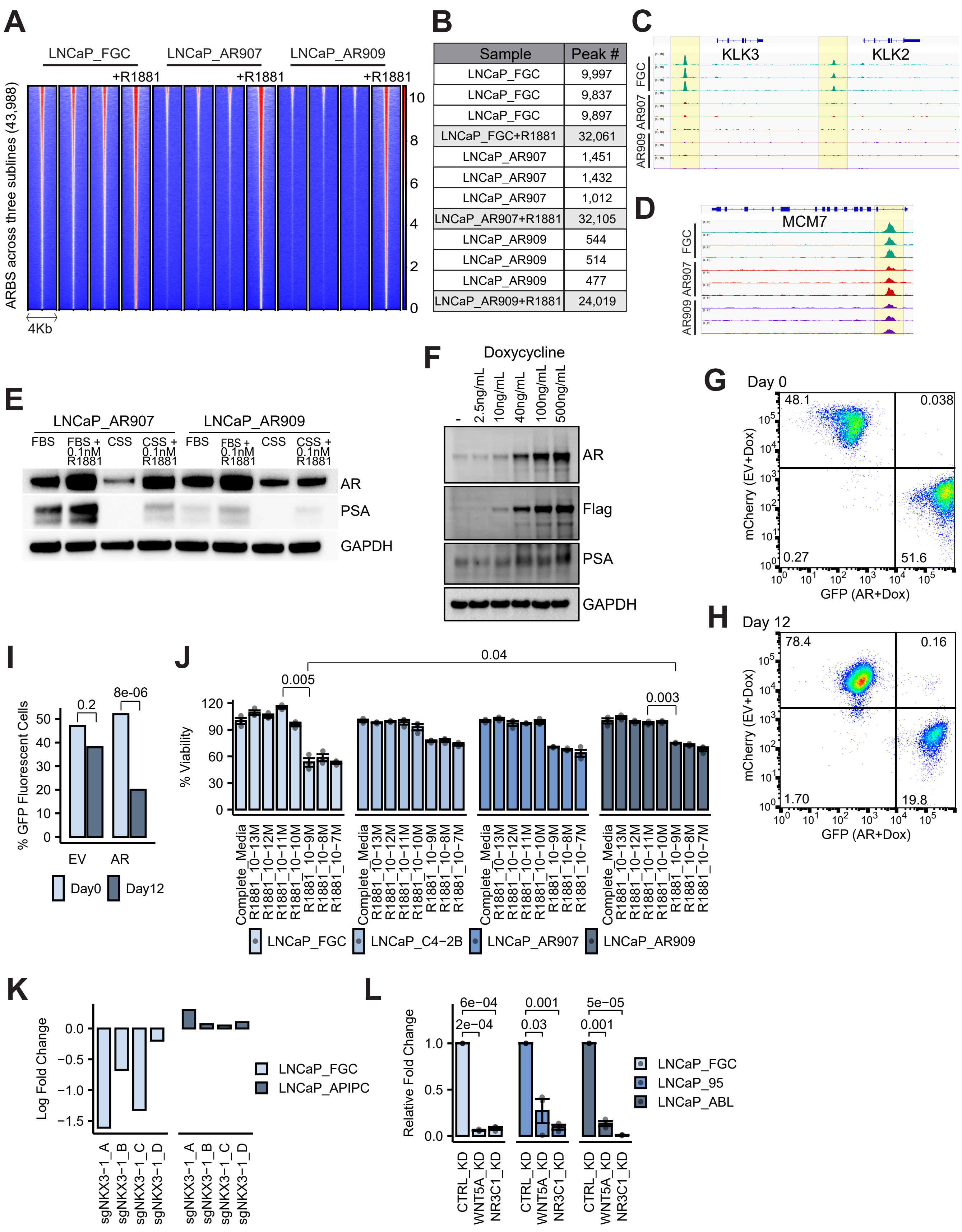

Sample	Peak #
LNCaP_FGC	9,997
LNCaP_FGC	9,837
LNCaP_FGC	9,897
LNCaP_FGC+R1881	32,061
LNCaP_AR907	1,451
LNCaP_AR907	1,432
LNCaP_AR907	1,012
LNCaP_AR907+R1881	32,105
LNCaP_AR909	544
LNCaP_AR909	514
LNCaP_AR909	477
LNCaP_AR909+R1881	24,019

Supplementary Figure S6. LNCaP sublines exhibit differential drivers and dependencies relevant for metastatic PC.

(A) ChIP-AR signal of LNCaP_FGC, LNCaP_AR-907, and LNCaP_AR-909 (with or without the addition of 10nM R1881in the standard growth medium) across the combined ARBS (36,318) among all listed samples.

(B) Summary table of ARBS peak numbers found in LNCaP_FGC, LNCaP_AR-907 and LNCaP_AR-909 under the designated growth condition.

(C) Integrative Genomics Viewer (IGV) of ChIP-AR peaks in LNCaP_FGC, LNCaP_AR-907, and LNCaP_AR-909 at KLK3 and KLK2 chromatin loci.

(D) IGV of ChIP-AR peaks in LNCaP_FGC, LNCaP_AR-907, and LNCaP_AR-909 at MCM7 chromatin loci.

(E) Immunoblot assessment of AR, PSA and GAPDH protein in LNCaP_AR907 and LNCaP_AR909 strains under conditions of steady state growth in 10% fetal bovine serum (FBS), androgen deprivation in charcoal-stripped medium (CSS) and androgen supplementation.

(F-I) Effects of DOX-inducible AR overexpression in LNCaP_FGC cells on the indicated protein expressions and cell growth, as assessed by competition assay. (F) induction of AR and PSA with increasing concentrations of doxycycline; (G) Plot of AR-induced GFP cells versus AR-control mCherry cells at Day 0 and (H) Day 12 after Doxycyline treatment; (I) Quantification shown for the change in the percent of AR-overexpressing GFP^{+} cells compared to vector-control cells for P0 and P1 (Day12) time points. Fisher's exact test of proportions of GFP+ vs. GFP- populations BH adjusted P values shown on plot.

(J) Assessment of cell viability by cell-titer glo assay in LNCaP strains across a range of androgen concentrations (n=3 per line.) Groups compared by unpaired t-tests with BH adjusted P values shown on plot.

(K) Depletion of individual sgRNA guides targeting *NKX3.1* in whole genome CRISPR screens of LNCaP_FGC and LNCaP_APIPC cells. Shown are relative log fold change assessed from MAGeCK analysis.

(L) Knockdown of WNT5A and NR3C1/GR in LNCaP_FGC, LNCaP_95 and LNCaP_ABL by shRNA. Shown are transcript levels by relative fold change against non-targeting control shRNA in each line (n=3 per line and condition). Groups compared by unpaired t-tests with BH adjusted P values shown on plot.

Supplementary Figure S7

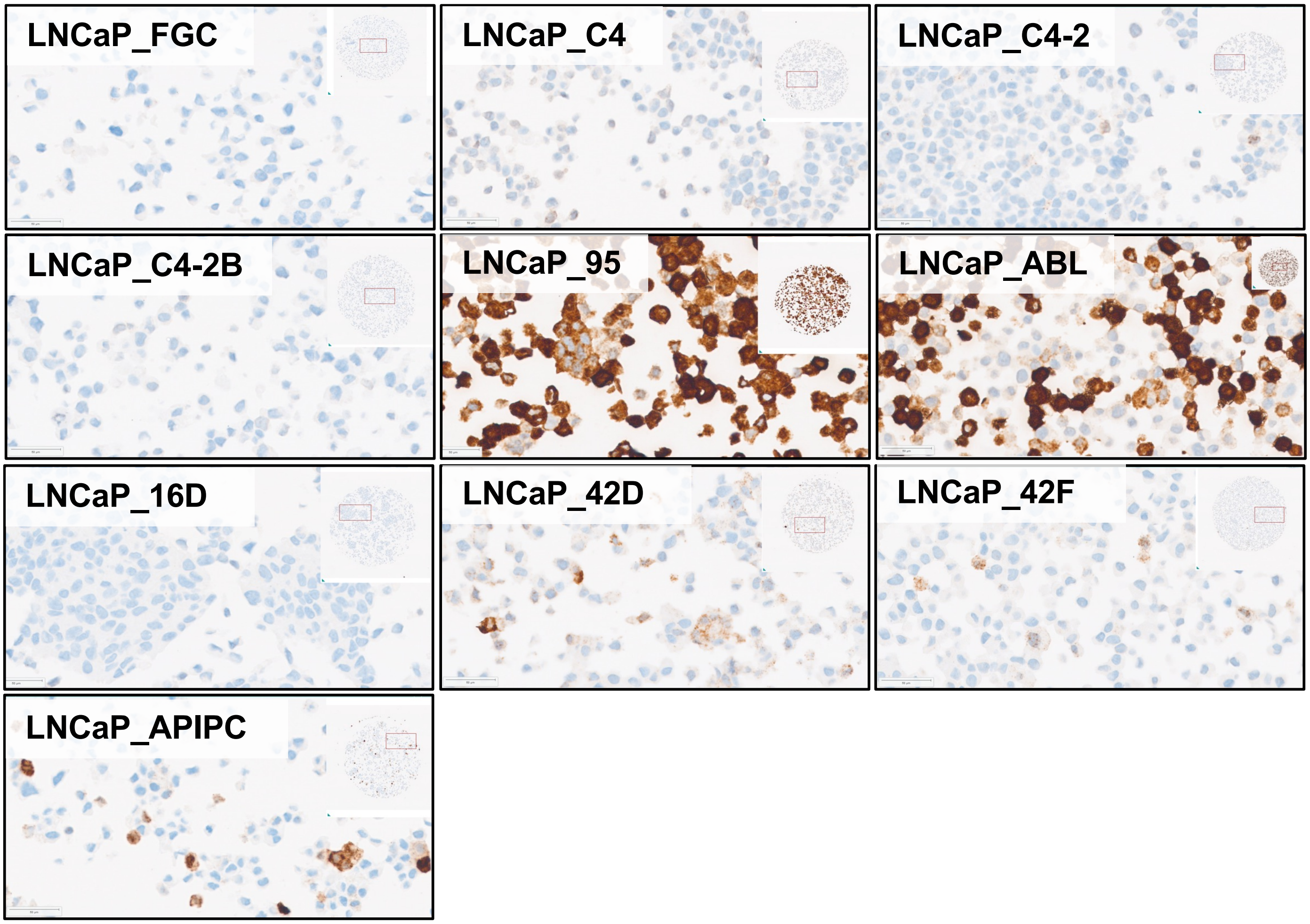

Supplementary Figure S7. Immunohistochemical assessment of NR3C1/GR protein expression in LNCaP strains.

SUPPLEMENTARY METHODS

Cell Lines and Culture

LNCaP_FGC, (ATCC CRL-1740), LNCaP_C4 (CRL-3313), LNCaP_C4-2 (CRL-3314) and LNCaP_C4-2B (CRL-3315) were obtained from the American Type Tissue Culture (ATCC) repository. LNCaP_ABL and LNCaP_95 cells were provided by S.Plymate, University of Washington, Seattle, WA. LNCaP_shAR cells were provided by Paul Rennie, University of British Columbia, Vancouver, BC. LNCaP_APIPC cells were derived from LNCaP_shAR cells and described in Bluemn et al(1). LNCaP_16D, LNCaP_42D and LNCaP_42F cells were provided by Dr. Amina Zoubeidi, University of British Columbia, Vancouver, BC. LNCaP_AR907 and LNCaP_AR909 cells (also known as LNCaP/AR) were provided by Dr. Charles Sawyers, Memorial Sloan Kettering Cancer Center, New York, NY. All cell lines were maintained at 37°C in a 5% CO_2 incubator. Each line or strain were grown in the recommended steady state growth medium detailed by the supplier or investigator: LNCaP_FGC, LNCaP_C4, LNCaP_C4-2, LNCaP_C4-2B, LNCaP_shAR, LNCaP_16D, LNCaP_AR907, and LNCaP_AR909 were grown in RPMI1640 supplemented with 10%FBS and 1%Pen/Strep antibiotic. LNCaP_ABL and LNCaP_95 were grown in RPMI1640 and 10% Charcoal Dextran Stripped FBS (CSS) and 1% Pen/Strep. LNCaP_42D and LNCaP_42F were grown in RPMI1640 supplemented with 10% FBS, 10μM Enzalutamide and 1% Pen/Strep. LNCaP_shAR/pATK were maintained in RPMI1640 with 5% FBS and 1% Pen/Strep, 2.5 mg/mL blasticidin, 1 mg/ml puromycin, and 25 mg/ml Zeocin. LNCAP_APIPC were grown in RPMI1640 with 5% CSS, 1%Pen/Strep, 2.5 mg/mL blasticidin, 1 mg/ml puromycin, 25 mg/ml Zeocin, and 1 mg/mL doxycycline. All cell lines were tested negative for mycoplasma. All cell lines were authenticated by STR genotyping and all matched to LNCaP_FGC.

Genome Sequencing and Analysis

<u>Genome Sequencing</u>: DNA was extracted using the Qiagen Puregene kit from each cell line grown at steady state conditions. DNA concentration was measured by Qubit.. DNA was submitted to the MedGenome whole genome sequencing service. Briefly, library construction libraries was prepared using the TruSeq DNA PCR-Free kit TruSeq DNA kit and sequenced using an Illumina NovaSeq 6000 sequencer (Illumina, San Diego, CA) using the S4 flow cell (300 cycles) with paired-end reads of 150 base pairs in read length.

<u>Sequence alignment.</u> DNA sequencing reads were aligned to the hg38 human genome using BWA-MEM (version 0.7.17). Alignments were then sorted and indexed using samtools (version 1.10), and duplicates were marked using picardtools MarkDuplicates (version 2.18.29). Finally, alignments were subjected to base quality score recalibration using GATK's BaseRecalibrator and ApplyBQSR (GATK version 4.1.8.1). Read counts and the percent of properly mapped reads were collected using CollectAlignmentSummaryMetrics, and CollectWgsMetrics was used to gather mean coverage (GATK version 4.1.8.1).

<u>Somatic mutation analysis</u>. For WGS and WES data, single nucleotide variation (SNV) and insertion and deletion (Indel) calling was performed using MuTect2 (GATK version 4.1.8.1) in tumor-only mode with a germline resource. MuTect2 for WES samples was run with default settings, and WGS samples were run with a base quality score threshold minimum of 6, minimum base quality score of 1 for base calling, and minimum base quality for pileup value of 1. All SNV and Indel calls were annotated using ANNOVAR (release 20200607). SNV and Indel inclusion were determined using various filters for allele frequency, pathogenicity, and read counts. SNVs and Indels were included if they possessed an allele frequency above 0.1 in Exome Aggregation Consortium (ExAC) and Genome Aggregation Database (gnomAD) as well as a variant allele frequency (VAF) > 0.1, total filtered depth > 10, and allelic depth of tumor > 3. Variants were considered pathogenic if they had annotations either within COSMIC (a COSMIC ID) or were annotated as "pathogenic" or "likely pathogenic" in InterVar and ClinVar. For relevant analyses, curated lists of prostate cancer-relevant gene BED files with latest hg38 coordinates were used to filter mutation calls. Validity of SNV and Indel calls was determined through manual curation using IGV (version 2.16.2).
Marker exonic single nucleotide variants (SNVs) that were unique to LNCaP lines were identified by first filtering to single nucleotide mutations that were annotated as exonic. Mutations were first filtered to

remove SNVs observed in more than one LNCaP line. Mutations with VAF <0.2 and depth <25 were removed. Next, mutations in Mutect filtered_all.vcf files were used to consider non-PASS mutation calls in removing candidate marker SNVs that were not unique. Finally, samtools mpileup v1.17 was used to count alt supporting reads for candidate marker SNVs in all lines and confirm that each SNV was present in only one line using a threshold of 2 or more reads. For this analysis LNCaP_AR907 and LNCaP_AR909 were grouped together where only SNVs called in both LNCaP_AR907 and LNCaP_AR909 were considered as markers for both lines because there were too few distinguishing exonic SNVs for LNCaP_AR907/LNCaP_AR909 lines when evaluated separately.

Somatic copy number alteration analysis. For WGS samples (n = 15), the standard tumor-only workflow of TITAN (v1.15.0) was used. Read counts were computed at 10 kb bins, and heterozygous SNPs were identified on the each LNCaP sample using SAMtools mpileup function. Read coverage in each bin across the genome was corrected for GC content and mappability biases for tumor samples using ichorCNA (v0.3.4). Copy number analysis was performed using TitanCNA, and results were curated for optimal ploidy and purity estimates. The analysis was performed without a matched normal sample using the tumor only pipeline: https://github.com/GavinHaLab/TitanCNA_SV_WGS.

TitanCNA typically relies on germline heterozygous sites, but in tumor-only analysis, these heterozygous SNP sites are determined from the tumor sample itself. Because LNCaP samples have nearly pure tumor cellularity, regions of homozygosity (such as LOH) will not have heterozygous sites. Therefore, we performed a post-processing step to rescue coverage information at these LOH regions from ichorCNA results.

Mutation signature analysis. SNVs that passed gnomAD and ExAC filters (frequency of < 0.1 in both) were formatted into VCF format per sample with a 5-column format of chromosome, genomic coordinate, sample ID, reference allele, and alternate allele. All sample VCFs were passed together through SigProfilerExtractor (v1.1.23) to extract mutation signatures and generate a matrix of mutation counts for 96 Single Base Substitution (SBS) patterns. The presence and proportions of COSMIC signatures were determined using SigProfilerAssignment (v0.0.33) with the matrix output from the previous step as input. Within the Analyze_comsic_fit() function, genome build was set to "GRCh38", cosmic_version set as "3", context_type set as "96", and input_type = "matrix". In order to best capture relevant signatures within the samples, SBS42 and signatures corresponding to signature subgroups "UV_signatures" and "Artifact_signatures" were excluded from the analysis. Signature proportions were determined by dividing the number of SNVs for each signature by the total number of SNVs per sample.

Clonality and phylogenentic analyses. Mutation clonality analysis was performed using coding SNVs that had a total allelic depth ≥ 10, a variant allelic depth in the tumor ≥ 3, and an allele frequency below 0.1% in the Exome Aggregation Consortium (ExAC) and Genome Aggregation Database (gnomAD). Additionally, mutations with a variant allele frequency (VAF) between 0.0-0.45 and 0.60-0.80 were retained. Mutations were considered functional if they were either annotated within COSMIC (a COSMIC ID) or as "pathogenic" or "likely pathogenic" in ClinVar, or had a deleterious status in at least one database such as SIFT, LRT, FATHMM, or MutationTaster, which are available as part of filter-based annotation details from ANNOVAR(2). This resulted in 24,282 mutations meeting these criteria.

PyClone-VI(version 0.1.1)(3) was used to analyze the filtered SNV mutations. Default parameters were used with a beta binomial model, ten restarts, and up to 40 clusters. The PyClone-VI results were used as inputs for the lineage inference method LICHeE(version 1.0) to elucidate clonal phylogenies. LICHeE(version 1.0)(4) to generate clonal lineage phylogenies. LICHeE was run in cellular prevalence mode with an error threshold of 0.1. Clusters with fewer than 200 mutations were removed to reduce overfitting and ensure robust tree construction. All other parameters were set to default. The top-ranking lineage tree from LICHeE was retained. Visualization of the clonal composition for each LNCaP substrain was created using the cloneMap R package.

LNCaP subline lineage analysis was also performed using neighbor-joining phylogenetic tree construction. All coding mutations, including splicing mutations, were used in this analysis, which was encoded as a binary status. The genetic distance was computed using Hamming/Manhattan distance, followed by neighbor-joining using the R package ape (v5.8-1). Nodes were rotated to have order the clades; edge lengths

were not shown to scale.

Structural variation analysis. For all samples, SvABA (https://github.com/walaj/svaba v134), Manta (https://github.com/Illumina/manta v1.6.0), and GRIDSS (https://github.com/PapenfussLab/gridss 2v0.0.1) were used in germline SV calling mode with default parameters to detect SV events. SVs were combined together using sv-merge (https://github.com/bankhead3/sv-merge) with 200bp/0.8 reciprocal overlap matching criteria to remove duplicates and match calls from SvABA, Manta, and GRIDSS. Merged calls were annotated using Titan combineSVABAandTITAN to be classified into SV types: deletion, inversion, tandem duplication, balanced and unbalanced rearrangements. Inter-chromosomal events were classified as translocations. Breakends transecting with genes were annotated using ensembl GRCh38.p12 HGNC symbols. SVs were matched between samples using 200bp/0.8 reciprocal overall matching criteria. SVs were filtered to meet the following criteria: 1) Support by two or more SV callers. 2) Not overlapping with ENCODE blacklist V2 or within +/-1Mb of AR. 3) Support by spanning/split reads in at least one LNCaP sample. 4) intra-chromosomal span size ≥10kb or inter-chromosomal or fold back inversion. SVs meeting criteria 1-4 are summarized in the manuscript. SVs meeting criteria 1-3 are included in Table S3. Circos plots were generated using RCircos v1.2.2 using Table S3 "Structural_variation_events" and svabaTitan annotated copy number files.

Gene Alteration by copy-number. Copy number segments were excluded if their cellular fraction was lower than 0.8, except for those which were determined as copy neutral or had a copy number greater than 4. Gene annotation was based on coordinates of known protein coding genes from GenCode release 30 (GRCh38.p12). For each gene, its corresponding copy number was assigned to the copy number value and LOH status of the TitanCNA-ichorCNA segment with the greatest overlap with it. Gene-level copy number was normalized based on ploidy of the respective sample. Then, copy number status of each gene was categorized based on a ratio between the calculated gene-level copy number and ploidy of the sample. Ratio thresholds were assigned the following classifications: (i) Amplification: Normalized gene-level copy number is greater than or equal to 2.5; (ii) Shallow Deletion (1 copy deleted): Normalized gene-level copy number is between 0.75 and 1; (iii) Deletion (2 copies deleted): Normalized gene-level copy number is between 0.5 and 0.75; (iv) Deep Deletion (3 copies deleted): Normalized gene-level copy number is between 0.25 and 0.5; (v) Homozygous Deletion: Normalized gene-level copy number is 0; (vi) LOH: Any of the above events, with an additional LOH status determined by if the Minor Copy Number of the corresponding segment is 0; (vii) Copy Neutral: Normalized gene-level copy number is between 0.95 and 1.05.

RNA Sequencing and Analysis

RNA Sequencing: Total RNA was extracted and DNAse-treated using the AllPrep kit (Qiagen). RNA concentration, purity, and integrity was assessed by NanoDrop (Thermo Fisher Scientific) and Agilent TapeStation. RNA-seq libraries were constructed from 1 ug total RNA using the Illumina TruSeq Stranded mRNA LT Sample Prep Kit according to the manufacturer's protocol. Barcoded libraries were pooled and sequenced by Illumina NovaSeq 6000 generating 50 bp paired end reads. Sequencing reads were mapped to the hg38 human genome and mm10 mouse genomes using STAR v2.7.3a (5). Gene fusions were mapped and quantitated using STAR-Fusion and Arriba v2.4.0. Quantification of AR splice variants was performed as previously described (6). All subsequent analyses were performed in R.

Transcript abundance and pathway analyses: Gene level abundance was quantitated using GenomicAlignments(7). Differential expression between groups was assessed using limma (8), filtered for a minimum expression level using the filterByExpr function with default parameters prior to testing, and using the Benjamin-Hochberg false discovery rate (FDR) adjustment. Genome-wide gene expression results were ranked by their limma statistics and used to conduct Gene Set Enrichment Analysis (GSEA)(9) to determine patterns of pathway activity utilizing the curated pathways from within the MSigDB v2023.1.Hs. Single sample enrichment scores were calculated using GSVA(10) with default parameters using genome-wide log2 FPKM values as input. A phenotypically diverse set of 32 samples, including LNCaP_FGC, LNCaP sublines, NCI-H660, VCaP, and 22Rv1 were used to calculate GSVA scores.

Single Cell RNA-Sequencing (scRNAseq)

scRNAseq sequencing. To collect single cell suspension, cells were trypsinized and washed once in PBS and re-suspended in cell culture media followed by hemocytometer cell count to ensure cell viability above 70%. The cell suspension were loaded into Chromium Next GEM Chip G (10x Genomics , PN#1000120) with 3′ v.3.1 beads (10x Genomics , PN#2000164) and partitioning oil. Individual cells were barcoded using a 10× Chromium Controller (10x Genomics). RNA from the barcoded cells were subsequently reverse-transcribed and amplified with a Chromium Single Cell 3′ v3.1 Reagent Kit (10x Genomics, PN#1000123). Libraries were subsequently constructed from 25% of amplified cDNA traces from previous step with reagents from a Chromium Single Cell 3′ v3.1 Library Kit (10x Genomics, PN#1000190). The cDNA and library traces were quantitated and identified by Tapestation. Sequencing were performed with an Illumina NextSeq P3.

scRNAseq analysis. Raw FASTQ files were processed using CellRanger v6.0. Downstream analysis was performed using Seurat V5.0 (7). Initial QC filtering removed cells with: number genes < 200, number reads > upper outer fence, number reads > 10k, percent hemoglobin reads > 25, percent ribosomal reads > 50, percent mitochondrial reads > 15, quantiles of SCDS v1.16 doublet score and number reads > 90% (8). Seurat subset function was used to down-sample cell counts to be 1,850 and scuttle v1.12 was used to down-sample to 19k average reads per cell for comparability between the lines. Each line was processed separately using the standard Seurat workflow with 3,000 variable genes and cluster resolution set to 0.5.

Chromatin Immunoprecipitation Sequencing (ChIPseq)

Chromatin isolation, immunoprecipitation and sequencing. Cells were seeded in 15 cm² dishes in their respective growth medium and cultured for 3 days before performing ChIP experiments. Cells were fixed with 1% formaldehyde at room temperature for 10 minutes, and then quenched with 125 mM glycine. Cell nuclei were sequentially isolated using LB1 buffer (50 mM HEPES-KOH, pH 7.5, 140 mM NaCl, 1 mM EDTA, 10% glycerol, 0.5% NP-40, and 0.25% Triton X-100) followed by LB2 buffer (10 mM Tris-HCl, pH 8.0, 200 mM NaCl, 1 mM EDTA, and 0.5 mM EGTA). The nuclei were then lysed with ChIP lysis buffer (1% SDS, 5 mM EDTA, and 50 mM Tris-HCl, pH 8.1). Chromatin was sheared into ~300 bp fragments using the Bioruptor Plus Sonicator (Diagenode).

Immunoprecipitation was performed using an anti-AR antibody (ab108341). The precipitated protein-DNA complexes were reverse cross-linked overnight at 65°C. The following day, both input and ChIP DNA were purified using the QIAquick PCR Purification Kit (Qiagen, 28104). ChIP-seq libraries were prepared using the ThruPLEX DNA-Seq Prep Kit (Takara Bio, R400675). Next-generation sequencing (100 bp, paired-end) was conducted on an Illumina NovaSeq 2000 at the Fred Hutch Genomics Core.

ChIPseq Analysis. Fastq files were aligned to hg38 using the Burrows-Wheeler Aligner(11) version 0.7.17-r1188 using the BWA-mem function. Samtools(12) was used to filter for alignments with a MAPQ score of at least 30. PCR duplicates were marked using Picard MarkDuplicates v2.24.1 (Picard: https://broadinstitute.github.io/picard/) and then removed using Samtools. Peaks were called using MACS3 v3.0.0(13). Called peaks had the ENCODE blacklisted regions removed(14). For the correlation analysis, deduplicated BAM files from replicates were first quantified with multiBamSummary (deepTools v3.5.4(15)), and Spearman correlation coefficients were calculated with the plotCorrelation tool. Heatmaps of read coverage around genomic features of interest were produced using the computeMatrix and plotHeatmap commands in deepTools (v3.5.4). All IGV screenshots presented were captured using IGV v2.19.1(16).

Assay for Transposase-Accessible Chromatin using sequencing (ATACseq)

Nuclei Isolation and ATAC sequencing: Cultured cells were freshly dissociated with TrypLE at 37°C and washed with ice-cold PBS. Nuclei isolation and transposition was performed using the OMNI-ATACseq protocol (1) with some few adjustments (17). Briefly, 200, 000 cells were pelleted at 500 rcf for 5 minutes in a pre-chilled (4°C) fixed-angle centrifuge. Supernatant was carefully discarded in a 2-step method using a P1000 pipette followed by a P200 pipette. Pelleted cells were then resuspended in 100 µL ice-cold lysis buffer comprising ATACseq resuspension buffer (A-RSB; 10 mM Tris-HCl, 10 mM NaCl, 3 mM $MgCl_2$ in nuclease free H2O) supplemented with NP-40, tween-20 and digitonin at final concentrations of 0.1%,

0.1% and 0.01% respectively. After 3 minutes of lysis reaction on ice, 1ml of ice-cold wash buffer (A-RSB supplemented with tween-20; 0.1% final concentration) was added and the tubes inverted to mix. The nuclei were then pelleted by centrifugation, and suspension carefully discarded (as previously described). For transposition, nuclei were resuspended in a 50 µL transposition mix (2x TD buffer, PBS, 0.01% digitonin, 0.1 % tween 20, nuclease-free H20 and TDE1Tn5 transposase enzyme). Transposition was performed at 37°C for 30 minutes in a thermomixer with shaking at 850 rpm. Transposed DNA was cleaned up using the MinElute® PCR purification kit. Libraries were generated as previously described, double-sized selected using AMPure XP beads and eluted in EB buffer. For sequencing, 16 libraries were pooled together and sequenced pair-end in a NextSeq P3.

ATAC-Seq Data Analysis. Fastq files were aligned to human genome build hg38 using bowtie2 v2.4.2 (18). PCR duplicates were marked and removed using Picard MarkDuplicates v2.24.1 (https://broadinstitute.github.io/picard/). Peaks were called using Genrich v0.6.1 in ATAC-Seq mode (https://github.com/jsh58/Genrich?tab=readme-ov-file#atacseq). Called peaks had the ENCODE blacklisted regions removed(14). Differential analysis was performed using DiffBind 3.10.1(19).

A set of reproducible peaks was identified for each sample by merging peaks observed in 2 or more replicate samples with a reciprocal overlap of 0.9. For analyses across samples (PCA, consensus clustering), peaks were combined and merged together across samples. Peak replicates counts were summed for each sample. For PCA, sample counts were transformed using VST and the top 5000 most variable peaks were used. ConsensusClusterPlus v1.64 (20)was used to identify robust groups of LNCaP lines using the 5000 most variable peaks across 13 LNCaP lines with pItem = 0.9, reps = 1000, maxK = 10, and remaining parameters set to default. Visual inspection of the Delta area plot indicated the 6 cluster solution was optimal. DESeq2 v1.40.2 (21) was used to identify signature peaks that were differentially accessible using replicate counts for each cluster group and between select lines and LNCaP_FGC using a threshold of log2FC > 3 and q-value < 0.05. Homer v4.11 was used to annotate peaks and perform motif enrichment analysis using findMotifsGenome.pl with size = 250, mknown = vertebrate known.motifs, and remaining parameters set to default(22). Motif enrichment comparison plot was constructed by rank ordering transcription factors (TFs) by Homer enrichment p-value for each sample, calculating a rank difference between both samples, and only plotting TFs that were significantly enriched in at least one of the samples (q-value < 0.05). Peaks were annotated with ChiPseeker v1.4.0 and TxDb.Hsapiens.UCSC.hg38.knownGene v3.18.0 using a promoter region of -3kbp to 1kbp. For the signature peak enhancer versus promoter comparison, peaks with distal intergenic or intronic annotation were labeled as enhancers, peaks with any promoter annotation were labeled as promoters and a Fisher's Exact test was used to infer significance.

Protein Isolation and Immunoblotting

Protein was collected from adherent tissue culture cells after first rinsing them with PBS. They were directly lysed with a cell lysis buffer containing: 1.5 M Urea, 1% SDS, 1% NP-40, 2% Tween20, 250 nM NaCl in PBS and supplemented with a 1x protease inhibitor cocktail. Protein was quantified per protocol using a bicinchoninic acid assay. Normalized cell lysates were loaded onto a 4-12% NuPAGE Bis-Tris gel and run in MOPS buffer. Protein was transferred onto nitrocellulose membranes using a semi-dry transfer apparatus and Tris/CAPS buffer. Immunoblots were probed with primary antibodies targeting AR (Abcam, ab133273), PSA/KLK3 (Cell Signaling, #5365), and GAPDH (GeneTex, GTX627408).

Cell Manipulations: Gene Knockdowns, Knockouts and Growth Assays

short hairpin RNA (shRNA) gene knockdown: Mission shRNA (cloned into lentiviral vector pLKO.1-puro) targeting against human WNT5A (TRCN0000296083) and human GR (TRCN0000245004) were purchased from Sigma-Aldrich. An empty vector pLKO.1-puro from Addgene (plasmid#8453) was used as a negative control. 293T cells were co-transfected with shRNA and the second-generation lentiviral packaging plasmids pSPAX2 and pMD2.G using Lipofectamine 2000 (Invitrogen 11668019). 24h post-infection, cell medium was changed to the fresh one. 72h of transfection, virus- containing medium was collected and incubated with LentiX concentrator (Takara 631232) at 4^0C for overnight concentration before infecting target cells. Cells were selected with puromycin for 5-7 days. Knockdown of target genes were

confirmed by western blot analysis.

CRISPR/Cas9 mediated gene deletion: sgRNA oligonucleotides targeting human AAVS1, AR, NKX3-1 were designed and constructed according to the Broad Institute guideline https://portals.broadinstitute.org/gppx/crispick/public. Annealed oligoes were cloned into lentiCRISPRv2 purchased from Addgene#52961. 293T cells were used for Lentivirus production containing using the second-generation lentiviral packaging plasmids pSPAX2 and pMD2.G. LNCaP_FGC cells and substrains were transduced with virus and selected for 5-7 days with puromycin before performing experiments.

Cell survival/growth assays: Cell viability was determined by trypan blue exclusion. 6 X10^4 cells were seeded in each well of a 24-well plate. 48h later, cells were harvested and counted with a cell viability analyzer Vi-Cell^{TM}XP (Beckman Coulter). For analysis, percent viability of cells targeted with WNT5A and GR shRNA was normalized to that of cell targeted with control shRNA. GFP-tagged LNCaP sublines were infected with sgRNA lentivirus. 48h later, selection with puromycin (1.5 ug/ml) was started that continued for 5-7 days. After selection, 2 X10^3 cells were seeded in a 96-well plate (Corning) in 200ul growth media and grown for 5 days in Incucyte incubator. Cells were read once in 24h using the bright- field and GFP channel with 4X objective. Cell media were changed in regular interval.

For growth competition analyses, GFP-NLS expressing LNCaP-FGC, C4-2B and 16D cells were infected with lentiviruses harboring paired sgRNAs against *AR*, *NKX3-1,* or control *AAVS1* locus and selected with puromycin for ~5-7 days. Cells were counted using a hemocytometer and 2 x 10^3 cells were seeded per well of a 96-well plate and imaged once every day for 5 days on an Incucyte S3 Live Cell Analysis Instrument (Sartorius) (n=3).

For cell viability analysis by trypan blue exclusion assays, 6 x 10^4 cells were seeded per well of a 24-well plate. Cells were harvested 48hr later and counted with a cell viability analyzer- Vi-CellTMXP (Beckman Coulter). For analysis, percent viability of cells targeted with WNT5A, and NR3C1/GR shRNAs was normalized to that of cell targeted with control shRNA (n=3). Differences were determined by unpaired t-test in R with Benjamini–Hochberg multiple testing correction using a threshold of 0.05 for significance.

Transcript abundance assays: Total RNA was extracted from cells using Qiagen RNeasy kit (Qiagen 74004) following the manufacturer's protocol and quantified by Qubit (Thermo Q10211). cDNA was synthesized by reverse transcription using with superscript II RT kit (Thermo 18064014). qPCR was performed using Power SYRB Green Mix (Thermo 4367660) in 384-well plate (10µL reactions) and run on a QuantStudio 6 Flex (Thermo 4485691) (have to check). Relative expression was standardized (ΔCT) to GAPDH and quantified using ΔΔCT, generating log2(fold change). PCR primers for WNT5A: Forward - AGGGCTCCTACGAGAGTGCT and Reverse - GACACCCCATGGCACTTG; NR3C1 (GR) Forward - TCT GAA CTT CCC TGG TCG AA and Reverse GTG GTC CTG TTG TTG CTG TT; GAPDH Forward – TCCTGCACCACCAACTGCTTAG and Reverse – AGTGGCAGTGATGGCATGGACT. Differences were determined by unpaired t-test in R with Benjamini–Hochberg multiple testing correction using a threshold of 0.05 for significance .

Cell competition assay. LNCaP-FGC cells were infected with lentiviruses harboring nuclear localization signal (NLS) containing GFP or mCherry fluorescent reporters and sorted using Sony SH800S Flow cytometer to generate the respective syngeneic cohorts. LNCaP-FGC GFP-NLS cells were then infected with lentiviruses harboring paired sgRNAs against either *AR*, *NKX3-1,* or control *AAVS1* locus, selected with puromycin and mixed in ~1:1 ratio, for each of the conditions, with LNCaP-FGC mCherry-NLS cells, similarly infected and selected only for sg*AAVS1*. The respective data points for the subsequent time points for mCherry or GFP were collected on Sony SH800S Flow cytometer, analyzed using FlowJo software and plotted for the respective GFP populations on GraphPad Prism software (n=3). Differences were determined by unpaired t-test in R with Benjamini–Hochberg multiple testing correction using a threshold of 0.05 for significance.

Pooled whole genome CRISPR deletion screen.

LNCaP_FGC cells and LNCaP_APIPC cells were transduced with lentivirus encoding the human Brunello CRISPR KO pooled library (Addgene # 73179) and selected with puromycin for 5 days to enrich for the

positive transductants. Infections were performed to achieve a representation of at least 500 cells per sgRNA per replicate (500X; ~40 x 10^6 cells per replicate). Cells were split upon confluence, re-seeded back in culture and maintained at a density of 500X over six population doublings (PD6). After PD6, ~40 x 10^6 cells per replicate were harvested and processed for genomic DNA extraction, barcode amplification and next-generation sequencing, as described previously(23). Genes and guides depleted in LNCaP_FGC and LNCaP_APIPC cells were identified using MAGECK analysis(24). A count table was prepared using the count function. Sample groups were then compared using the test (RRE) comparison function. This produced sgRNA and gene tables. Gene tables were used to create comparison scatter plots in R version 4.2.0 using ggplot2 version 3.5.1.

Statistics.

Statistical analyses pertaining to each figure are included within the figure legends. For comparisons of distributions of categorical variables, we performed the Fisher's exact test using Benjamini–Hochberg multiple testing correction in R. Continuous variables were compared between groups with unpaired t-tests using Benjamini–Hochberg multiple testing correction in R. Pearson's correlation coefficient computed in R was used to study the relationships between variables shown in scatterplots. Growth curves were fit and compared by nonlinear regression in GraphPad Prism10.3.1.

Data availability

- The RNAseq data, ATACseq data and AR ChIPseq data are deposited in the GEO repository under the accession numbers GSE288591, GSE288843, GSE288878, GSE289031, and GSE289398. The WGS data are deposited under accession number: PRJNA1219540 in the sequence read archive.
- Original Western blot images and microscopy data reported in this paper will be shared by the lead contact upon request.

Code availability

Software code developed and the configurations for computational pipelines in this study can be accessed at https://github.com/GavinHaLab/LNCaP-subline-paper.

REFERENCES for METHODS

1. Bluemn EG, Coleman IM, Lucas JM, Coleman RT, Hernandez-Lopez S, Tharakan R, et al. Androgen Receptor Pathway-Independent Prostate Cancer Is Sustained through FGF Signaling. *Cancer Cell.* 2017;32(4):474-89 e6.
2. Wang K, Li M, and Hakonarson H. ANNOVAR: functional annotation of genetic variants from high-throughput sequencing data. *Nucleic Acids Res.* 2010;38(16):e164.
3. Gillis S, and Roth A. PyClone-VI: scalable inference of clonal population structures using whole genome data. *BMC bioinformatics.* 2020;21(1):571.
4. Popic V, Salari R, Hajirasouliha I, Kashef-Haghighi D, West RB, and Batzoglou S. Fast and scalable inference of multi-sample cancer lineages. *Genome Biol.* 2015;16(1):91.
5. Dobin A, Davis CA, Schlesinger F, Drenkow J, Zaleski C, Jha S, et al. STAR: ultrafast universal RNA-seq aligner. *Bioinformatics.* 2013;29(1):15-21.
6. Sowalsky AG, Figueiredo I, Lis RT, Coleman I, Gurel B, Bogdan D, et al. Assessment of Androgen Receptor Splice Variant-7 as a Biomarker of Clinical Response in Castration-Sensitive Prostate Cancer. *Clin Cancer Res.* 2022;28(16):3509-25.
7. Lawrence M, Huber W, Pages H, Aboyoun P, Carlson M, Gentleman R, et al. Software for computing and annotating genomic ranges. *PLoS Comput Biol.* 2013;9(8):e1003118.
8. Ritchie ME, Phipson B, Wu D, Hu Y, Law CW, Shi W, et al. limma powers differential expression analyses for RNA-sequencing and microarray studies. *Nucleic Acids Res.* 2015;43(7):e47.
9. Subramanian A, Tamayo P, Mootha VK, Mukherjee S, Ebert BL, Gillette MA, et al. Gene set enrichment analysis: a knowledge-based approach for interpreting genome-wide expression profiles. *Proc Natl Acad Sci U S A.* 2005;102(43):15545-50.

10. Hanzelmann S, Castelo R, and Guinney J. GSVA: gene set variation analysis for microarray and RNA-seq data. *BMC bioinformatics.* 2013;14:7.
11. Li H, and Durbin R. Fast and accurate short read alignment with Burrows-Wheeler transform. *Bioinformatics.* 2009;25(14):1754-60.
12. Danecek P, Bonfield JK, Liddle J, Marshall J, Ohan V, Pollard MO, et al. Twelve years of SAMtools and BCFtools. *Gigascience.* 2021;10(2).
13. Zhang Y, Liu T, Meyer CA, Eeckhoute J, Johnson DS, Bernstein BE, et al. Model-based analysis of ChIP-Seq (MACS). *Genome Biol.* 2008;9(9):R137.
14. Amemiya HM, Kundaje A, and Boyle AP. The ENCODE Blacklist: Identification of Problematic Regions of the Genome. *Sci Rep.* 2019;9(1):9354.
15. Ramirez F, Ryan DP, Gruning B, Bhardwaj V, Kilpert F, Richter AS, et al. deepTools2: a next generation web server for deep-sequencing data analysis. *Nucleic Acids Res.* 2016;44(W1):W160-5.
16. Robinson JT, Thorvaldsdottir H, Winckler W, Guttman M, Lander ES, Getz G, et al. Integrative genomics viewer. *Nat Biotechnol.* 2011;29(1):24-6.
17. Corces MR, Trevino AE, Hamilton EG, Greenside PG, Sinnott-Armstrong NA, Vesuna S, et al. An improved ATAC-seq protocol reduces background and enables interrogation of frozen tissues. *Nat Methods.* 2017;14(10):959-62.
18. Langmead B, and Salzberg SL. Fast gapped-read alignment with Bowtie 2. *Nat Methods.* 2012;9(4):357-9.
19. Ross-Innes CS, Stark R, Teschendorff AE, Holmes KA, Ali HR, Dunning MJ, et al. Differential oestrogen receptor binding is associated with clinical outcome in breast cancer. *Nature.* 2012;481(7381):389-93.
20. Wilkerson MD, and Hayes DN. ConsensusClusterPlus: a class discovery tool with confidence assessments and item tracking. *Bioinformatics.* 2010;26(12):1572-3.
21. Love MI, Huber W, and Anders S. Moderated estimation of fold change and dispersion for RNA-seq data with DESeq2. *Genome Biol.* 2014;15(12):550.
22. Duttke SH, Chang MW, Heinz S, and Benner C. Identification and dynamic quantification of regulatory elements using total RNA. *Genome Res.* 2019;29(11):1836-46.
23. Nyquist MD, Corella A, Mohamad O, Coleman I, Kaipainen A, Kuppers DA, et al. Molecular determinants of response to high-dose androgen therapy in prostate cancer. *JCI insight.* 2019;4(19).
24. Li W, Xu H, Xiao T, Cong L, Love MI, Zhang F, et al. MAGeCK enables robust identification of essential genes from genome-scale CRISPR/Cas9 knockout screens. *Genome Biol.* 2014;15(12):554.
