# Supplementary material for: Multiomic assessments of LNCaP and derived cell strains reveal determinants of prostate cancer pathobiology
Source: J Clin Invest. 2025 Sep 16;135(22):e194727. doi: 10.1172/JCI194727 (PMC12618073; doi:10.1172/JCI194727)
Supplement: Unedited blot and gel images [file jci-135-194727-s097.pdf]

194727-JCI-RG-1_Bose et al

Uncropped/unedited images for Figure S6F
(boxed area represent the cropped regions for final figure)

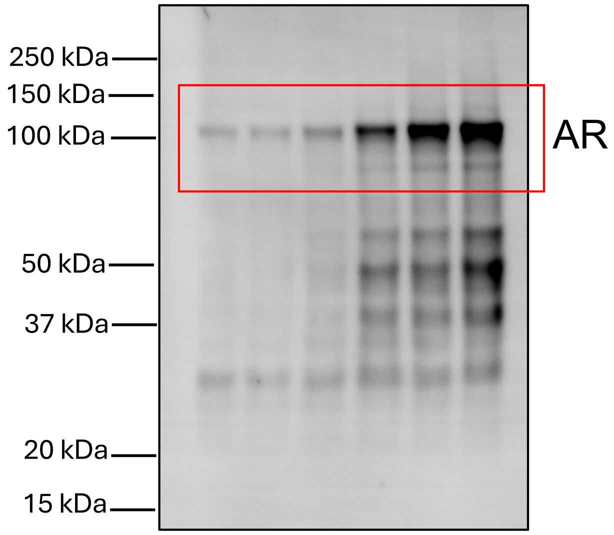

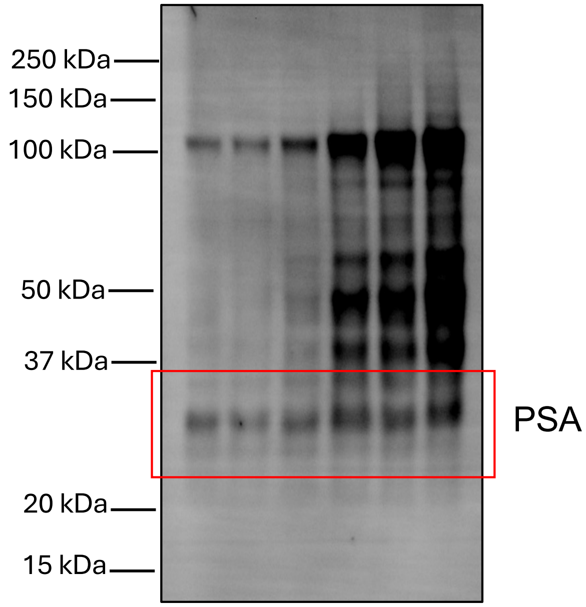

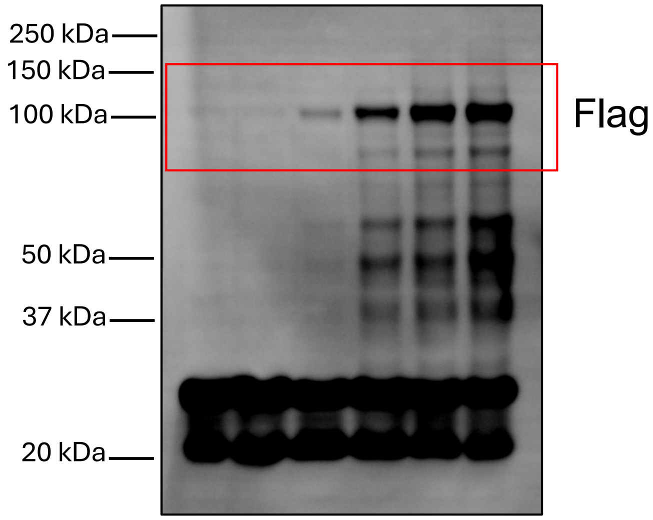

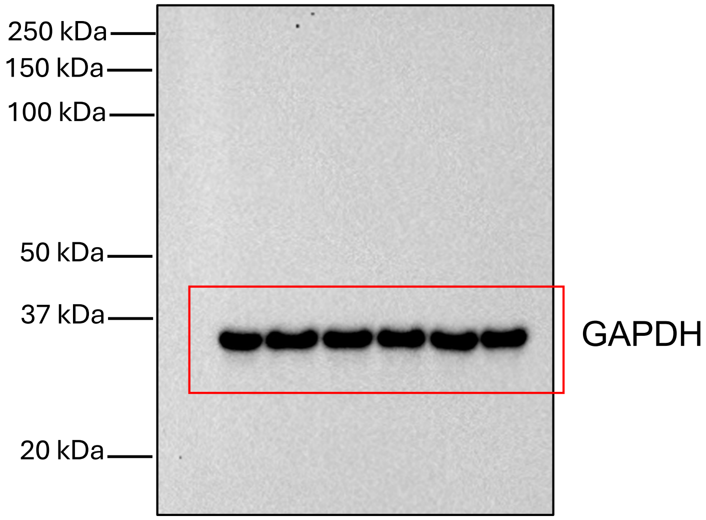

Antibodies used:
Anti-AR_(D6F11) XP® Rabbit mAb #5153_Cell Signaling Technology
Anti-PSA/KLK3_(D6B1) XP® Rabbit mAb #5365_Cell Signaling Technology
Anti-Flag_Monoclonal ANTI-FLAG® M2 Mouse #F1804_Millipore Sigma
Anti-GAPDH_Purified anti-GAPDH Antibody Rat #607902_BioLegend

194727-JCI-RG-1_Bose et al

Uncropped/unedited images for Figure S6E
(boxed area represent the cropped regions for final figure)

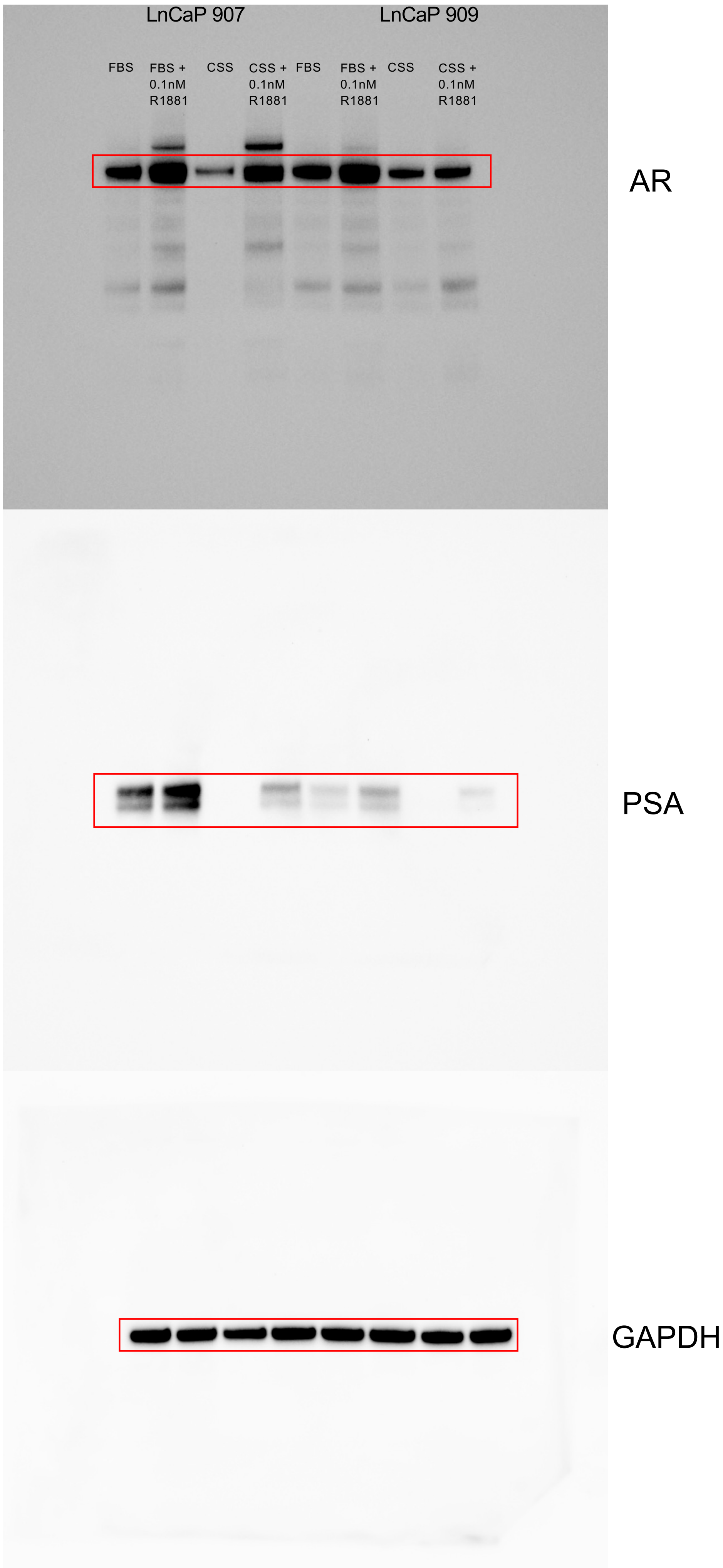

Antibodies used: Figure S6E
AR_Androgen Receptor antibody [EPR1535(2)] Abcam

PSA/KLK3_(D6B1) XP® Rabbit mAb #5365_Cell Signaling Technology

GAPDH_Purified anti-GAPDH Antibody Rat #607902_BioLegend

194727-JCI-RG-1_Bose et al

Uncropped/unedited images for Figure 6L
(boxed area represent the cropped regions for final figure)

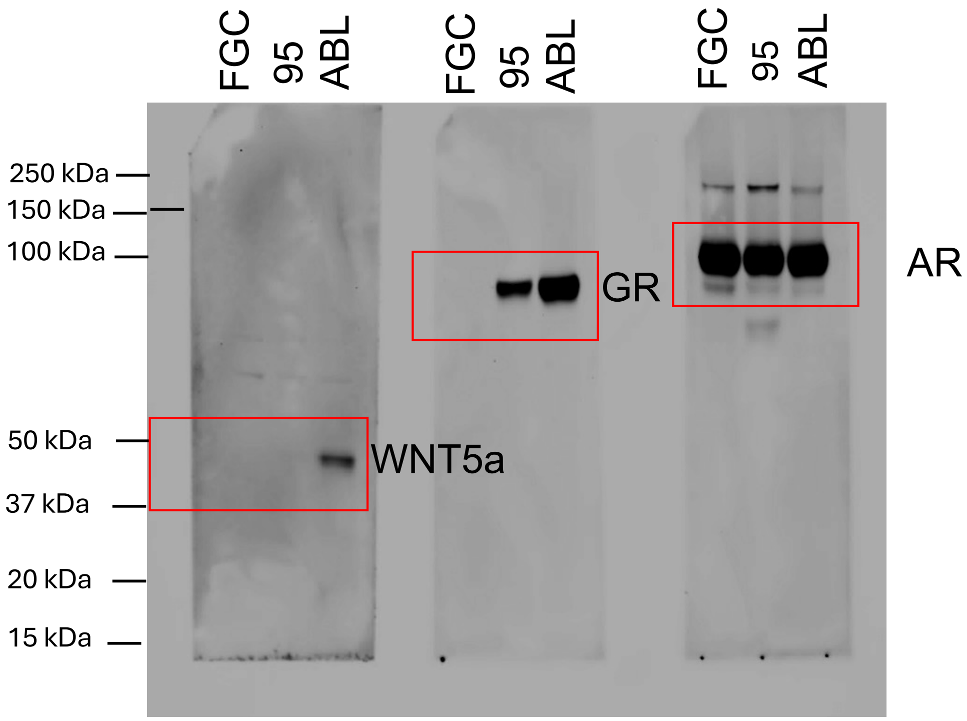

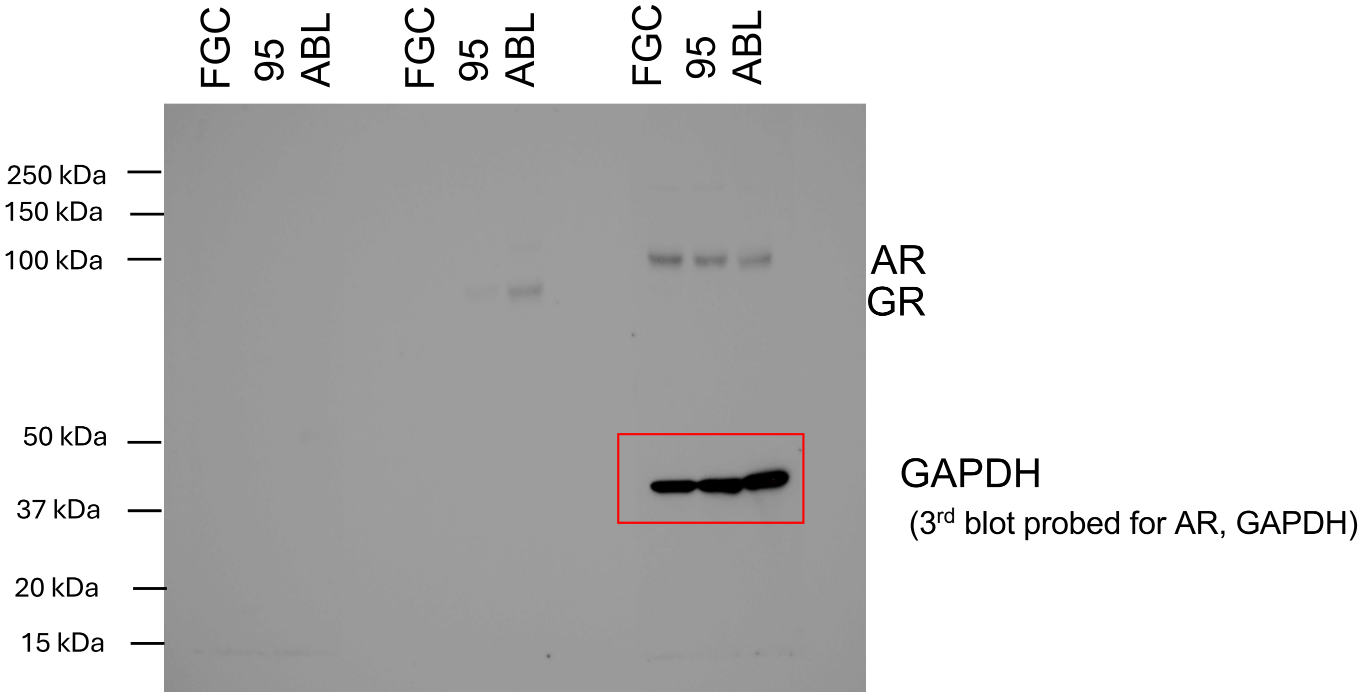

Antibodies used for Figure 6L:
Anti-AR_(D6F11) XP® Rabbit mAb #5153_Cell Signaling Technology
Anti-GR_(D6H2L) XP® Rabbit mAb #1204_ Cell Signaling Technology
Anti_Wnt5a/b (C27E8) Rabbit mAb# 2530S _ Cell Signaling Technology
Anti-GAPDH (GT239) Mouse mAb# GTX 627408 _ Genetex

Antibodies used for Figure 6L:
Anti-AR_(D6F11) XP® Rabbit mAb #5153_Cell Signaling Technology
Anti-GR_(D6H2L) XP® Rabbit mAb #1204_ Cell Signaling Technology
Anti_Wnt5a/b (C27E8) Rabbit mAb# 2530S _ Cell Signaling Technology
Anti-GAPDH (GT239) Mouse mAb# GTX 627408 _ Genetex
